# Multifunctional Fe_3_O_4_ @ Au core/shell nanostars: a unique platform for multimode imaging and photothermal therapy of tumors

**DOI:** 10.1038/srep28325

**Published:** 2016-06-21

**Authors:** Yong Hu, Ruizhi Wang, Shige Wang, Ling Ding, Jingchao Li, Yu Luo, Xiaolin Wang, Mingwu Shen, Xiangyang Shi

**Affiliations:** 1 State Key Laboratory for Modification of Chemical Fibers and Polymer Materials, College of Chemistry, Chemical Engineering and Biotechnology, Donghua University, Shanghai 201620, People’s Republic of China; 2 Shanghai Institute of Medical Imaging, Department of Interventional Radiology, Zhongshan Hospital, Fudan University, Shanghai 200032, People’s Republic of China; 3 College of Science, University of Shanghai for Science & Technology, Shanghai 200093, People’s Republic of China

## Abstract

We herein report the development of multifunctional folic acid (FA)-targeted Fe_3_O_4_ @ Au nanostars (NSs) for targeted multi-mode magnetic resonance (MR)/computed tomography (CT)/photoacoustic (PA) imaging and photothermal therapy (PTT) of tumors. In this present work, citric acid-stabilized Fe_3_O_4_/Ag composite nanoparticles prepared by a mild reduction route were utilized as seeds and exposed to the Au growth solution to induce the formation of Fe_3_O_4_ @ Au core/shell NSs. Followed by successive decoration of thiolated polyethyleneimine (PEI-SH), FA *via* a polyethylene glycol spacer, and acetylation of the residual PEI amines, multifunctional Fe_3_O_4_ @ Au NSs were formed. The designed multifunctional NSs possess excellent colloidal stability, good cytocompatibility in a given concentration range, and specific recognition to cancer cells overexpressing FA receptors. Due to co-existence of Fe_3_O_4_ core and star-shaped Au shell, the NSs can be used for MR and CT imaging of tumors, respectively. Likewise, the near infrared plasmonic absorption feature also enables the NSs to be used for PA imaging and PTT of tumors. Our study clearly demonstrates a unique theranostic nanoplatform that can be used for high performance multi-mode imaging-guided PTT of tumors, which may be extendable for theranostics of different diseases in translational medicine.

Molecular imaging (MI) provides the means to study *in vivo* processes that have tremendous potential applications in biomedical research and clinical diagnosis^1^,^2^,^3^. Although each modality imaging has its own merits, no single technique is capable of giving complete information in disease diagnosis due to its intrinsic drawbacks in terms of sensitivity, spatial and temporal resolution, multiplexing capability, and response time^4^,^5^,^6^. Among many types of MI technologies, magnetic resonance (MR) imaging has been generally considered as one of the most powerful noninvasive imaging techniques owing to its great spatial resolution and tomographic capabilities^7^,^8^,^9^,^10^. Computed tomography (CT) affords better spatial and density resolution than other imaging techniques^11^,^12^. In addition, photoacoustic (PA) imaging is a non-invasive imaging technique with high resolution and provides fast, quantitative, volumetric measurement with deep tissue penetration capability^13^,^14^. However, the spatial resolution of MR imaging is lower than that of CT imaging, while the soft tissue contrast of CT imaging is lower than that of MR imaging. Meanwhile, PA imaging is still at the phases of basic research for now and have not been widely used in clinical applications. Therefore, combination of these three diagnostic modalities is expected to be able to overcome some serious restrictions encountered by each MI technique when used alone, leading to much more accurate disease diagnosis, in particular cancer. Besides precision cancer diagnosis, it is desirable to perform simultaneous treatment of cancer. Among many different cancer therapy approaches, photothermal therapy (PTT) has attracted great interest in recent years due to the advantage that the light-induced heating, as a non-invasive strategy, is able to ablate cancerous cells without damaging surrounding normal tissues^15^,^16^,^17^,^18^.

Recent advances in nanotechnology exhibit vast potential to generate various platforms that can be used for cancer theranostics^19^,^20^,^21^. Among the used nanomaterials, iron oxide (Fe_3_O_4_) nanoparticles (NPs), due to their ability to shorten the T_2_ relaxation time of their surrounding water protons and biocompatibility, can be used as T_2_ negative contrast agents for MR imaging^10^,^22^. On the other hand, gold (Au) NPs, owning to the higher atomic number of Au than that of iodine for iodinated CT contrast agents (e.g., Omnipaque), have been explored as CT imaging agents^23^,^24^. For accurate dual mode MR/CT imaging applications, Fe_3_O_4_/Au composite nanoparticles (CNPs) has been designed^25^,^26^. For example, Cai *et al*. synthesized Fe_3_O_4_/Au CNPs with the assistance of dendrimers that can be used for MR/CT imaging of animal organs/tissues^25^ and tumors *in vivo*^27^. Zhu *et al*.^20^ reported the preparation of Au-Fe_3_O_4_ heterostructured NPs for dual mode MR/CT imaging of liver in an intravital model. It is known that Au NPs with a particular shape such as nanostars (NSs)^28^,^29^,^30^, nanorods^31^,^32^,^33^, nanoflowers^21^,^34^, or nanocages^35^,^36^ are able to exhibit strong localized surface plasmon resonance (SPR) absorbance in near infrared (NIR) region for PTT of cancer cells^15^,^31^,^37^,^38^. Furthermore, Au NPs with these specific shapes can be used for PA imaging through the absorption of a pulsed light and the detection of the resultant ultrasonic (US) wave profile by special transductor^13^,^14^. For example, Nie *et al*.^13^ demonstrated that gold NSs were able to be simultaneously used for PTT and PA imaging of tumors. Accordingly, in order to develop a unique platform that can be used for simultaneous multimode imaging and PTT of tumors, it is desirable to integrate Fe_3_O_4_ NPs with specific shaped Au NPs.

In our previous study, we have shown that Fe_3_O_4_ @ Au NSs can be formed by exposing Fe_3_O_4_ @ Ag seed particles to Au growth solution, and can be functionalized *via* polyethyleneimine (PEI)-mediated covalent conjugation reaction for *in vivo* MR/CT imaging and PTT of tumors *via* intratumoral administration of the particles^39^. However, this study was limited to use hyaluronic acid as a targeting ligand and to just use intratumoral injection as an administration route. Furthermore, the PA imaging potential of the developed Fe_3_O_4_ @ Au NSs has not been explored. Therefore, it is still challengeable to develop multifunctional Fe_3_O_4_ @ Au NSs using different approaches for multimode imaging-guided PTT of tumors.

In this present work, a unique multifunctional nanoplatform based on folic acid (FA) - modified Fe_3_O_4_ @ Au NSs were designed for tri-mode MR/CT/PA imaging and PTT of tumors. Fe_3_O_4_/Ag composite particles were first synthesized by sodium borohydride (NaBH_4_) reduction of Ag(I) ions on the surface of citric acid (CA)-coated Fe_3_O_4_ NPs formed *via* a mild reduction route according to the literature^10^,^40^,^41^. The as-prepared Fe_3_O_4_/Ag composite particles were utilized as seeds and exposed to the aqueous Au growth solution to induce the formation of Fe_3_O_4_ @ Au NSs. Thereafter, the obtained Fe_3_O_4_ @ Au NSs were decorated with thiolated PEI (PEI-SH) *via* Au-S bond. Finally, the PEI-coated Fe_3_O_4_ @ Au NSs were sequentially conjugated with FA through a polyethylene glycol (PEG) spacer *via* the PEI amine-enabled conjugation chemistry. This was followed by acetylation of the remaining PEI amines (Fig. 1a). The formed multifunctional FA-modified Fe_3_O_4_ @ Au (Fe_3_O_4_ @ Au-PEI.Ac-PEG-FA) NSs were exhaustively characterized. Their hemocompatibility, cytocompatibility, specific recognition to FA receptor (FAR)-overexpressing cancer cells, and potential for multi-mode MR/CT/PA imaging and PTT of tumors were evaluated in detail.

## Results

### Formation and characterization of the Fe_3_O_4_ @ Au-PEI.Ac-PEG-FA NSs

According to our previous work^10^, Fe_3_O_4_ NPs were synthesized *via* a mild reduction route for targeted *in vivo* MR imaging of tumors. This facile mild reduction strategy enabled the generation of Fe_3_O_4_ NPs with ultrahigh r_2_ relaxivity. In this study, by virtue of the same approach, we synthesized CA-stabilized Fe_3_O_4_ NPs (Fe_3_O_4_ @ CA) and further created Fe_3_O_4_/Ag seed particles by NaBH_4_ reduction of Ag(I) in the presence of the Fe_3_O_4_ @ CA NPs according to the literature^41^. The formed Fe_3_O_4_/Ag seed particles are quite uniform in size (Supplementary Fig. S1a) with an average size of 9.3 nm (Fig. S1b). The apparent absorption peak at 400 nm can be ascribed to the SPR peak of Ag component (Fig. S1c), confirming the formation of Fe_3_O_4_/Ag seed particles. Likewise, the existence of both Ag and Fe in the energy dispersive spectrum (Fig. S1d) also confirmed the formation of the Fe_3_O_4_/Ag seed particles. The composition of the Fe_3_O_4_/Ag seed particles was further quantified using ICP-OES and the molar ratio of Fe_3_O_4_/Ag was measured to be 1.9:1.

By exposing the Fe_3_O_4_/Ag seeds to the Au growth solution, star-shaped Fe_3_O_4_ @ Au were generated. The NIR-absorbance feature of the NSs was validated by UV-vis spectroscopy (Supplementary Fig. S2). By optimizing the concentration of the chemicals, Fe_3_O_4_/Au NSs with a desirable NIR-absorption feature were obtained using an Au growth solution containing 2.4 mM HAuCl_4_, 0.08 mM AgNO_3_, 5.2 mM AA. The formed CTAB-stabilized Fe_3_O_4_ @ Au NSs display unique star-shaped spikes with a narrow size distribution (Supplementary Fig. S3). In order to make the NSs be functionalized, the NSs were copiously washed to remove the surfactant CTAB, and modified with PEI-SH *via* Au-S bond formation according to our previous work^39^. The PEI-SH synthesized was characterized by ^1^H NMR to have 15.3 thiol groups per PEI (Supplementary Fig. S4a). The generated PEI-stabilized Fe_3_O_4_ @ Au (Fe_3_O_4_ @ Au-PEI) NSs with a large amount of primary amines were further grafted with COOH-PEG-FA, which was characterized to have 0.8 FA moieties per PEG by ^1^H NMR (Fig. S4b). This FA modification onto the NSs *via* a PEG spacer is expected to endow the NSs with high affinity to cancer cells overexpressing FAR^19^. Finally, the synthesized Fe_3_O_4_ @ Au-PEI-PEG-FA NSs were subjected to an acetylation reaction to neutralize the residual PEI surface amines in order to improve their cytocompatibility^42^.

Zeta potential and hydrodynamic size of NSs produced in each step were measured to confirm their surface modification (Supplementary Table S1). Clearly, Fe_3_O_4_ @ Au-PEI NSs possess a quite positive surface potential (+31.4 mV) due to the surface modification of PEI with a large amount of amines. After the successive modification of COOH-PEG-FA and acetylation reaction, the surface potentials of the Fe_3_O_4_ @ Au-PEI-PEG-FA and Fe_3_O_4_ @ Au-PEI.Ac-PEG-FA NSs were measured to be +28.3 and +14.4 mV, respectively. The decreased surface potential for both NSs validated the success of the modification of COOH-PEG-FA and acetylation reaction when compared to that of the Fe_3_O_4_ @ Au-PEI NSs. Likewise, the hydrodynamic sizes of the Fe_3_O_4_ @ Au-PEI-PEG-FA (226.1 nm) and Fe_3_O_4_ @ Au-PEI.Ac-PEG-FA (224.2 nm) NSs are larger than that of the Fe_3_O_4_ @ Au-PEI NSs (211.3 nm), suggesting the successful surface modification of the NSs. Additionally, the hydrodynamic size of the final Fe_3_O_4_ @ Au-PEI.Ac-PEG-FA NSs was also occasionally measured within a time period of 15 days (Supplementary Fig. S5). We show that the hydrodynamic size of NSs does not display any obvious fluctuation, suggesting their laudable colloidal stability. Moreover, the long-term colloidal stability of the Fe_3_O_4_ @ Au-PEI.Ac-PEG-FA NSs was evaluated by exposing them to different media (water, PBS, and DMEM containing 10% FBS, respectively) for at least one month (Fig. S5 and inset). The particles do not precipitate, further confirming their long-term colloidal stability.

To quantify the conjugated PEI and COOH-PEG-FA on the surface of the Fe_3_O_4_ @ Au NSs, TGA were performed (Supplementary Fig. S6). At 700 °C, the PEI coating results in a weight loss of 4.8% for the Fe_3_O_4_ @ Au-PEI NSs, when compared with the CTAB-free Fe_3_O_4_ @ Au NSs. Further conjugation of COOH-PEG-FA affords the Fe_3_O_4_ @ Au-PEI-PEG-FA NSs with an increased weight loss of 12.4%. Therefore, the modified COOH-PEG-FA onto the NSs was deduced to be 7.6%.

**Figure 1:**
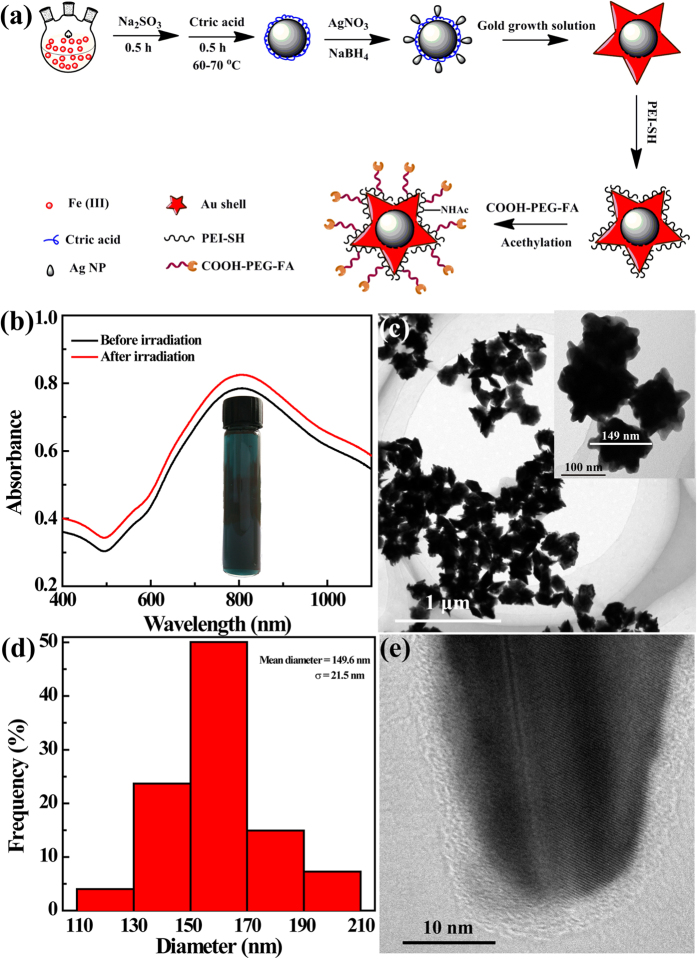
Synthesis and characterization of the Fe_3_O_4_ @ Au-PEI.Ac-PEG-FA NSs. (**a**) Schematic representation of the synthesis of the Fe_3_O_4_ @ Au-PEI.Ac-PEG-FA NSs. (**b**) UV-vis spectra of the aqueous solution of Fe_3_O_4_ @ Au-PEI.Ac-PEG-FA NSs before and after laser irradiation (inset shows the digital photo of the Fe_3_O_4_ @ Au-PEI.Ac-PEG-FA NSs in aqueous solution). (**c**) TEM image (inset is the high magnification TEM image), (**d**) size distribution histogram, and (**e**) high-resolution TEM image (only an Au spike is shown) of the Fe_3_O_4_ @ Au-PEI.Ac-PEG-FA NSs.

The optical property of the Fe_3_O_4_ @ Au-PEI.Ac-PEG-FA NSs was investigated by UV-vis spectroscopy (Fig. 1b). The dark blue color of NSs in aqueous solution was ascribed to the star-shaped Au shell coating on the particle surface (Fig. 1b, inset). An obvious SPR peak in the NIR region at 810 nm can be clearly seen, and the NIR absorption feature does not show obvious changes even when the NSs were irradiated with an 808 nm laser at 2.0 W/cm^2^ for 20 min. This suggests that the NSs display an excellent photothermal stability and also a great potential to use them for PTT of tumors. TEM was employed to observe the morphology and size of the Fe_3_O_4_ @ Au-PEI.Ac-PEG-FA NSs (Fig. 1c–e). We can clearly see that the NSs with Au shell coating onto the surface of Fe_3_O_4_ NPs possess well-defined star shape and a quite uniform size distribution (Fig. 1c). By measuring two maximal margins of the NSs, the mean diameter of NSs was estimated to be 149.6 ± 21.5 nm (Fig. 1d). As depicted in Fig. 1e, high-resolution TEM image reveals the clear lattices of the spike-like Au shell crystals and also a dense polymer coating on the outer surface of the NSs, which is relevant to the PEI modification and COOH-PEG-FA grafting. The elemental composition of Fe and Au in the Fe_3_O_4 _ @ Au-PEI.Ac-PEG-FA NSs was quantified by ICP-OES, and the Fe/Au molar ratio was measured to be 1:53.4.

### MR and CT phantom studies

**Figure 2:**
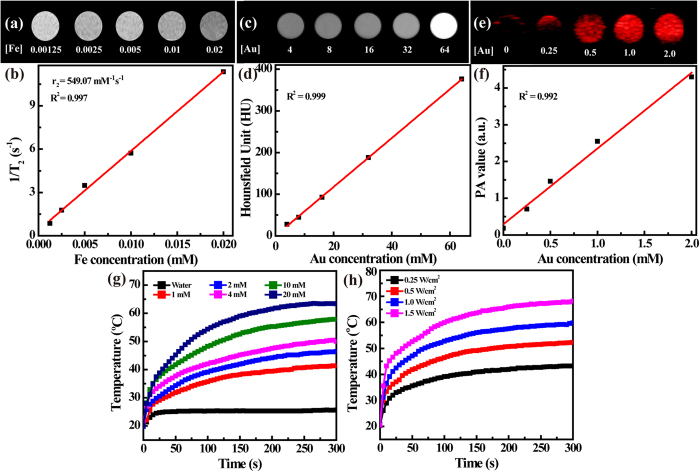
MR relaxometry, X-ray attenuation property, photoacoustic property and photothermal performance of the Fe_3_O_4_ @ Au-PEI.Ac-PEG-FA NSs. (**a**) T_2_-weighted MR images and (**b**) linear fitting of 1/T_2_ of the Fe_3_O_4_ @ Au-PEI.Ac-PEG-FA NSs as a function of Fe concentration. (**c**) CT images and (**d**) linear fitting of CT values (HU) of the Fe_3_O_4_ @ Au-PEI.Ac-PEG-FA NSs at an Au concentration of 4, 8, 16, 32, and 64 mM, respectively. (**e**) PA images and (**f**) linear fitting of PA values of the Fe_3_O_4_ @ Au-PEI.Ac-PEG-FA NSs at an Au concentration of 0, 0.25, 0.5, 1.0, and 2.0 mM, respectively. (**g**) Temperature change of water and water containing the Fe_3_O_4_ @ Au-PEI.Ac-PEG-FA NSs at varying Au concentrations (1, 2, 4, 10, and 20 mM, respectively) under an 808 nm laser irradiation with the same power density of 1.0 W/cm^2^ as a function of irradiation time. (**h**) Temperature change of a water solution containing the Fe_3_O_4_ @ Au-PEI.Ac-PEG-FA NSs at an Au concentration of 10 mM under an 808 nm laser irradiation with different power densities (0.25, 0.5, 1.0, and 1.5 W/cm^2^, respectively) as a function of irradiation time.

Fe_3_O_4_ NPs are generally used as MR contrast agents because of their capacity to shorten the T_2_ relaxation time of their surrounding water protons. From T_2_-weighted MR imaging, we can find that the generated Fe_3_O_4_ @ Au-PEI.Ac-PEG-FA NSs can gradually decrease the MR signal intensity of water with the increase of Fe concentration (Fig. 2a). By linearly fitting the T_2_ relaxation rate (1/T_2_) *versus* Fe concentration, the r_2_ relaxivity of the NSs was calculated to be 549.07 mM^−1^s^−1^ (Fig. 2b). The ultrahigh r_2_ relaxivity of the NSs should be due to the nature of the mild reduction approach used to synthesize Fe_3_O_4_ NPs, in agreement with the literature^10^. It seems that the Au shell coating does not appreciably affect the accessibility of water protons to the inner Fe_3_O_4_ NPs, due largely to the interstitial spaces between each Au spikes of the NSs.

On the other hand, CT phantom studies of the Fe_3_O_4_ @ Au-PEI.Ac-PEG-FA NSs were carried out to explore their potential for CT imaging (Fig. 2c). It can be clearly seen that, with the increase of Au concentration, the CT image of the NSs-containing aqueous suspension gradually brightens, well matching the quantitative measurement of their CT value (HU) *versus* Au concentration (Fig. 2d).

### Photoacoustic property and photothermal performance of the Fe_3_O_4_ @ Au-PEI.Ac-PEG-FA NSs

To explore the feasibility to employ the Fe_3_O_4_ @ Au-PEI.Ac-PEG-FA NSs for PA imaging, PA phantom studies were first carried out (Fig. 2e). Clearly, with the Au concentration of the NSs, the PA signal intensity gradually enhances, which correlates well with the quantitative PA signal intensity change *versus* Au concentration (Fig. 2f).

The photothermal behavior of the Fe_3_O_4_ @ Au-PEI.Ac-PEG-FA NSs was next investigated to unleash their potential to be used for PTT of tumors. The temperature variation of the the NSs-containing aqueous solution *versus* Au concentration (1–20 mM) was continuously monitored in real time after exposure to an 808 nm laser at 1.0 W/cm^2^ for 300 s (Fig. 2g). Clearly, the NSs are able to induce a positive temperature enhancement in a concentration-dependent manner. The temperature of the NS suspension can reach 63.3 °C at the Au concentration of 20 mM. For comparison, laser irradiation of pure water under the same conditions does not afford obvious temperature increase. At a given Au concentration of 10 mM, the NS suspension irradiated under an 808 nm laser at different output power densities (0.25–1.5 W/cm^2^) for 300 s was also monitored to check the temperature change (Fig. 2h). It is evident that the NSs are able to generate heat in a laser output power density-dependent manner. The temperature of the NS suspension rises to 68.5 °C at the highest laser output power density (1.5 W/cm^2^). These results indicate that the synthesized Fe_3_O_4_ @ Au-PEI.Ac-PEG-FA NSs were able to transform NIR laser into heat rapidly under laser irradiation. The photothermal conversion efficiency (η) of the NSs was calculated according to the literature^43^,^44^. Details can be seen in Supplementary information. The η of the NSs was calculated to be 88.9%, which is prominently higher than that of other major PTT agents^43^,^44^,^45^,^46^.

### Hemolytic and cytotoxicity assays

For biomedical applications, it is vital to evaluate the hemocompatibility and cytocompatibility of the prepared Fe_3_O_4_ @ Au-PEI.Ac-PEG-FA NSs. The hemolytic activity of the NSs was evaluated by hemolytic assay (Supplementary Fig. S8a). When compared to the positive water control, where obvious hemolytic behavior occurs, NSs at different Au concentrations do not display appreciable hemolysis effect, similar to the negative PBS control (inset of Fig. S8a). The hemolysis percentages of HRBCs exposed to NS solution at different Au concentrations were calculated to be 0.39%, 1.79%, 2.51%, and 4.39%, respectively, which are all less than the threshold value of 5%, indicating their excellent hemocompatibility in the given Au concentration range^47^,^48^.

The cytocompatibility of the Fe_3_O_4_ @ Au-PEI.Ac-PEG-FA NSs was assessed by MTT assay of viability of HeLa cell (Fig. S8b). Apparently, after cultivation of HeLa cells with NSs at an Au concentration of 0.2, 0.4, 0.8, 1.5, and 2.0 mM, respectively, for 24 h, the cell viability still keeps larger than 80%, suggesting their negligible cytotoxicity^19^,^47^. The cytocompatibility of the NSs was further validated by observing the morphology of HeLa cells (Supplementary Fig. S9). Clearly, the morphology of HeLa cells after treatment with the NSs in the given Au concentration range (0.2–2.0 mM) (Fig. S9b–f) is quite similar to that of HeLa cells treated with PBS (Fig. S9a). Taken together, the developed Fe_3_O_4_ @ Au-PEI.Ac-PEG-FA NSs display good hemocompatibility and cytocompatibility in the studied concentration range.

### *In vitro* cellular uptake assay and targeted MR and CT imaging of cancer cells

**Figure 3:**
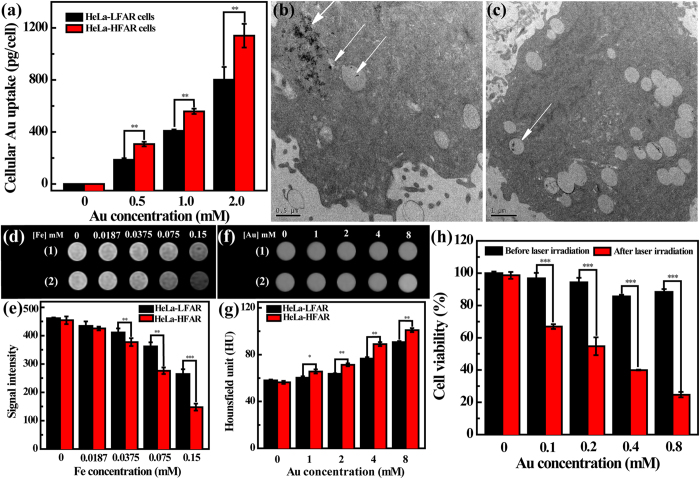
*In vitro* cellular uptake assay, targeted MR and CT imaging and photothermal ablation of HeLa cells. (**a**) The Au uptake by HeLa-HFAR and HeLa-LFAR cells after treated with the Fe_3_O_4_ @ Au-PEI.Ac-PEG-FA NSs at various Au concentrations for 4 h. TEM images of (**b**) HeLa-HFAR and (**c**) HeLa-LFAR cells after treated with the Fe_3_O_4_ @ Au-PEI.Ac-PEG-FA NSs for 4 h, respectively. (**d**) T_2_-weighted MR images, (**e**) MR signal intensity, (**f**) CT images, and (**g**) CT value of HeLa-HFAR and HeLa-LFAR cells treated with the Fe_3_O_4_ @ Au-PEI.Ac-PEG-FA NSs at varying Fe or Au concentrations for 6 h. (**h**) MTT assay of HeLa cell viability after treatment with the Fe_3_O_4_ @ Au-PEI.Ac-PEG-FA NSs in a given Au concentration range under an 808 nm laser irradiation for 5 min. 1 and 2 represent the HeLa-LFAR and HeLa-HFAR cells, respectively.

To verify the high affinity of the produced Fe_3_O_4_ @ Au-PEI.Ac-PEG-FA NSs to cancer cells overexpressing FAR, ICP-OES was performed to analyze the Au uptake by HeLa cells (Fig. 3a). Obviously, both HeLa-HFAR and HeLa-LFAR cells exhibit gradually enhanced Au uptake with the NS concentration. Under the same Au concentrations, the Au uptake in HeLa-HFAR cells was apparently higher than that in HeLa-LFAR cells (p < 0.01). This implies that the modified FA ligands onto the NSs enable specific targeting of the NSs to FAR-overexpressing cancer cells *via* FA-mediated pathway^49^,^50^,^51^. The specific uptake of the Fe_3_O_4_ @ Au-PEI.Ac-PEG-FA NSs was further evaluated by TEM imaging (Fig. 3b,c). Clearly, HeLa-HFAR cells treated with the NSs show the remarkable distribution into the cytoplasm of the cells (Fig. 3b). In contrast, only minimal NSs were able to be detected in HeLa-LFAR cells, which is associated to the nonspecific phagocytosis or diffusion *via* cell walls (Fig. 3c)^52^.

MR and CT imaging were also performed to further validate the specific targeting of the NSs to HeLa-HFAR cells. T_2_-weighted MR images of both HeLa-HFAR and HeLa-LFAR cells treated with the NSs become darker with the increase of Fe concentration (Fig. 3d). However, HeLa-HFAR cells show a more prominent MR signal intensity decrease under the same Fe concentrations than HeLa-LFAR cells. This was further validated by quantifying the MR signal intensity of HeLa-HFAR and HeLa-LFAR cells treated with the NSs (Fig. 3e). On the other hand, CT images and the quantitative CT values (HU) of the cells treated with the NSs (Fig. 3f,g) reveal that the CT contrast enhancement of HeLa-HFAR cells is much more obvious than that of HeLa-LFAR cells (p < 0.05) under the same Au concentrations, indicating that the FA renders the NSs with a targeting specificity to cancer cells that overexpress FAR. Overall, the developed Fe_3_O_4_ @ Au-PEI.Ac-PEG-FA NSs are able to be used for specific MR and CT imaging of FAR-overexpressing cancer cells *in vitro*.

### *In vitro* photothermal ablation of cancer cells

Inspired by the high-performance photothermal property and FA-enabled targeting specificity, we used the Fe_3_O_4_ @ Au-PEI.Ac-PEG-FA NSs for PTT of cancer cells *in vitro* (Fig. 3h). Apparently, HeLa cells after treatment with the NSs without laser irradiation are quite healthy with viability comparable to those treated with PBS (control). In sharp contrast, the viability of HeLa cells after treatment with the NSs and irradiated under an 808 nm laser (1.0 W/cm^2^) for 5 min markedly decreases even at the Au concentration as low as 0.1 mM (p < 0.001). With the increase of Au concentration, the NSs exert more prominent ablation effect on cancer cells and 75.3% of HeLa cells can be killed at an Au concentration of 0.8 mM.

The PTT of cancer cells using the Fe_3_O_4_ @ Au-PEI.Ac-PEG-FA NSs was further investigated by cell morphology observation (Supplementary Fig. S10). Clearly, the morphologies of HeLa cells treated with PBS (Fig. S10a,f) and the NSs alone at the Au concentrations of 0.1–0.8 mM are quite healthy (Fig. S10b–e). In sharp contrast, after treatment with the NSs plus laser irradiation (Fig. S10g–j), HeLa cells are detached and rounded even at the lowest Au concentration of 0.1 mM, indicating that the cells have undergone apoptosis. The cell morphology observation results corroborate the MTT viability assay data, confirming the excellent performance of the NSs for PTT of cancer cells *in vitro*.

### *In vivo* MR/CT/PA tri-mode imaging of a xenografted tumor model

**Figure 4:**
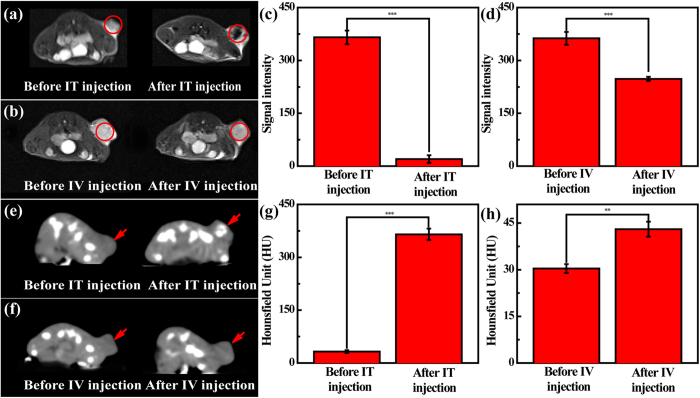
*In vivo* MR/CT imaging of a xenografted tumor model. (**a**,**b**) T_2_-weighted MR images, (**c**,**d**) MR signal intensity, (**e**,**f**) CT images, and (**g**,**h**) CT value of tumors before and at 0.5 h post IT (**a**,**c**,**e**,**g**) and at 6 h post IV (**b**,**d**,**f**,**h**) injection of the Fe_3_O_4_ @ Au-PEI.Ac-PEG-FA NSs ([Fe] = 1.31 mM, [Au] = 70 mM, 0.1 mL in PBS).

Next, the feasibility to use the Fe_3_O_4_ @ Au-PEI.Ac-PEG-FA NSs for tri-mode MR/CT/PA imaging of xenografted HeLa tumors *in vivo* were explored. As shown in Fig. 4a,b, the region of tumor becomes markedly dark at 0.5 h post intratumoral (IT) injection or at 6 h post intravenous (IV) injection. Quantitative MR signal intensity analysis show that the tumor MR signal intensity dramatically decreases from 256.6 to 20.1 and from 362.7 to 247.7 for IT and IV injection, respectively (p < 0.001) (Fig. 4c,d). This validates the use of the NSs for MR imaging of the tumors *in vivo.* To demonstrate the targeting specificity of the NSs, free FA-blocked HeLa tumor was also imaged by MR after IV injection under the same condition (Supplementary Fig. S11). Clearly, the tumor region displayed similar brightness at 6 h post IV injection to that before injection (Fig. S11a), which can be further confirmed by quantitative MR signal intensity analysis (Fig. S11b).

**Figure 5:**
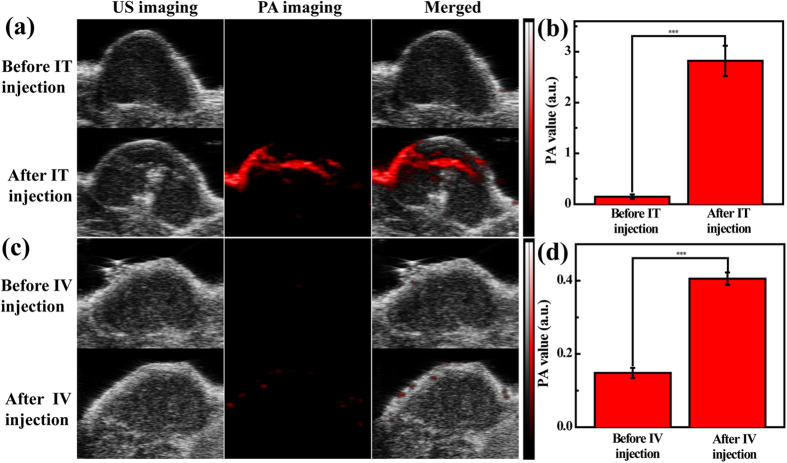
*In vivo* PA imaging of a xenografted tumor model. *In vivo* ultrasound/PA images (**a**,**c**) and PA value (**b**,**d**) of tumors before and at 0.5 h post IT ([Au] = 100 mM, 0.04 mL in PBS, **a**,**b**) and at 6 h post IV ([Au] = 100 mM, 0.1 mL in PBS, **c**,**d**) injection of the Fe_3_O_4_ @ Au-PEI.Ac-PEG-FA NSs.

We then tested the potential to use the NSs for CT and PA imaging of the HeLa tumor. For CT imaging, the brightness of tumor site increases at 0.5 h post IT injection or at 6 h post IV injection (Fig. 4e,f). This can be further validated by collecting the CT value of the tumor region (Fig. 4g,h). Clearly, the CT value of tumor site significantly increases from 32.6 to 364.9 HU at 0.5 h post IT injection and from 30.4 to 43.0 HU at 6 h post IV injection, respectively (p < 0.01). For PA imaging, once the NSs absorb NIR laser and transform it into heat, the ambient environment can be expanded under this heat and generate an ultrasound signal. We then performed both PA and ultrasound imaging of the tumors. As shown in Fig. 5a,c, intense PA signal of tumor area can be easily visualized after IT and IV injection of NSs into tumor-bearing mice. When compared to the PA intensity of tumor region before injection, the PA intensity is approximately 20 folds and 3 folds higher for IT injection (increase from 0.14 to 2.82) and IV injection (increase from 0.14 to 0.41), respectively (p < 0.001, Fig. 5b,d). The enhanced tumor MR/CT/PA imaging should be due to the modification of PEGylated FA onto the particle surfaces. On one hand (particularly for IV injection), the PEGylation modification of NSs enables them to escape from the uptake by the reticuloendothelial system (RES) and to accumulate in the tumor tissue *via* a passive targeting pathway based on enhanced permeability and retention (EPR) effect^53^; on the other hand, with the FA-mediated active targeting pathway as demonstrated in the *in vitro* studies, the NSs are able to specifically target the tumor tissue for highly effective tumor MR/CT/PA imaging.

### *In vivo* PTT of a xenografted tumor model

**Figure 6:**
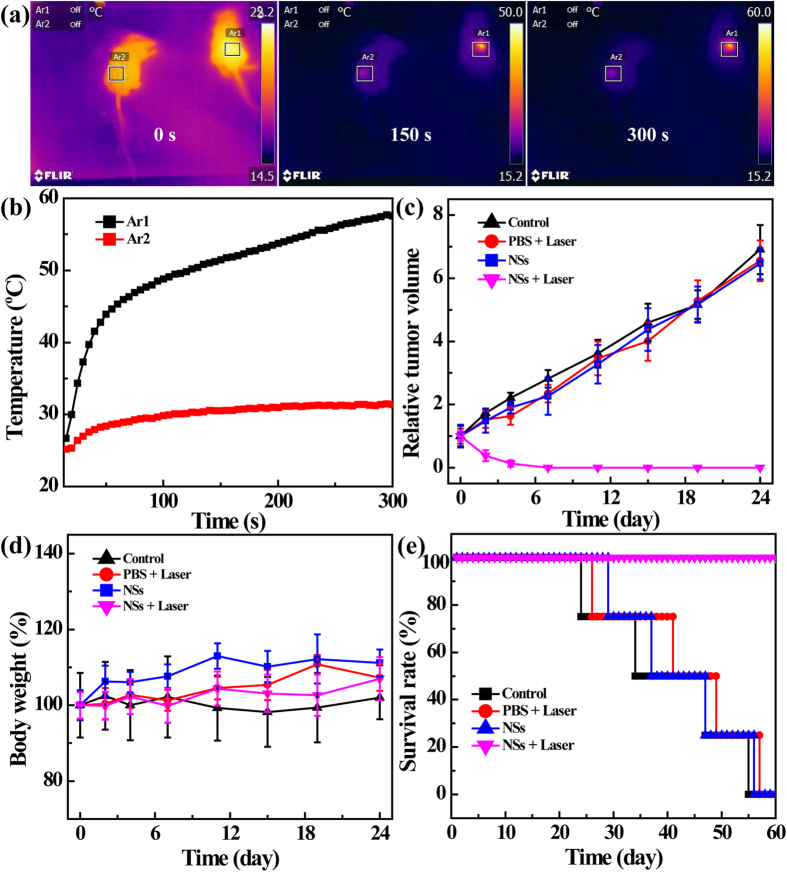
*In vivo* photothermal imaging and photothermal ablation of HeLa tumors. (**a**) Full-body photothermal images of HeLa tumor-bearing mice after IT injection with 0.1 mL PBS (control, the right mouse, indicated at region Ar2) or Fe_3_O_4_ @ Au-PEI.Ac-PEG-FA NSs dispersed in 0.1 mL PBS ([Au] = 20 mM, the left mouse, indicated at region Ar1), followed by exposure to an 808 nm laser with a power density of 1.0 W/cm^2^ at a time point of 0 min, 2.5 min, and 5 min, respectively. (**b**) The plot of temperature increase in tumor regions (Ar1 and Ar2) as a function of irradiation time. (**c**) The tumor volume change profiles, (**d**) body weight change, and (**e**) survival rate of tumor-bearing mice (n = 4) as a function of time posttreatment.

Next, the potential to employ the Fe_3_O_4_ @ Au-PEI.Ac-PEG-FA NSs for thermal imaging and PTT of tumors were tested (Fig. 6). Intravital thermal imaging of mice was conducted at different laser irradiation time periods using an infrared camera. As shown in Fig. 6a,b, the temperature of Region Ar1 (administrated with the NSs) rapidly increases by 24.7 °C within 150 s, and by 30.8 °C after 300 s of irradiation. In contrast, for Region Ar2 (administrated with PBS), a subtle temperature elevation was observed and the temperature increases just approximately 5 °C during the laser irradiation.

This high-temperature caused by NSs under laser irradiation is expected to ablate tumors. Next, we examined the tumor volume change of the mice after different treatments (Fig. 6c). The relative volumes of tumors at 24 days posttreatment for the groups of control (PBS), PBS + Laser, and NSs are approximately 6–7 times larger than that of the initial tumor. In sharp contrast, the treatment using NSs plus laser is able to completely ablate the tumors at 7 days postinjection of the NSs. This clearly illustrates the possibility to employ the NSs for highly efficient PTT of tumors. The PTT efficacy of tumors can be easily visualized by taking photos of the mice (Supplementary Fig. S12). The mice in the NSs + Laser group maintain healthy at 19 days posttreatment, and the completely ablated tumor region does not seem to recur in the studied time period, suggesting the excellent PTT efficiency of the Fe_3_O_4_ @ Au-PEI.Ac-PEG-FA NSs. In contrast, for the mice in the groups of Control, PBS + Laser, and NSs, the tumors grow bigger and bigger with the time. It should be noted that, during the experimental time period, mice with different treatments are able to maintain their body weights (Fig. 6d), suggesting that the treatments using the laser alone, the NSs alone, or the laser plus NSs are not toxic to the mice. Finally, the PTT efficacy of the tumors was evaluated by monitoring the survival rate of the mice after different treatments (Fig. 6e). Obviously, after treatment with the NSs plus laser, the mean survival rate of mice keeps 100% after 60 days. However, mice treated with PBS (Control group), PBS plus laser, and NSs alone survive with an average life-span of 40 days, 43 days, and 42 days, respectively.

### Histology examinations

**Figure 7:**
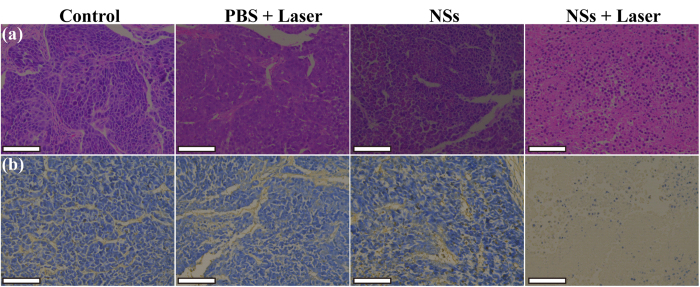
Histological examinations. (**a**) H&E and (**b**) TUNEL staining of tumor sections after the tumor mice were treated under different conditions. The scale bar shown in each panel of (**a**,**b**) represents 100 μm.

The PTT efficacy of tumors were further confirmed by histological examinations using H&E and TUNEL staining of harvested tumor sections (Fig. 7). H&E staining (Fig. 7a) reveals that the tumors either treated with PBS plus laser or the NSs alone exhibit normal HeLa tumor cell morphology, similar to those treated with PBS (control). But for tumors treated with the NSs plus laser, necrosis cells can be clearly seen in the whole section. Likewise, TUNEL staining (Fig. 7b) shows that only a very small amount of positive staining of apoptotic cells can be found in the sections of control, PBS + Laser, and NSs groups. In sharp contrast, after treatment with the NSs plus laser irradiation, the tumors display a huge amount of positive staining of apoptotic cells. Quantitative analysis data further reveal that the percentages of apoptotic cells in the tumors of the Control, PBS + Laser, NSs, and NSs + Laser groups are 1.5%, 3.3%, 1.3%, and 95.0%, respectively (Supplementary Fig. S13).

## Discussion

Generally, Fe_3_O_4_/Au CNPs could be synthesized *via* various routes and utilized for dual mode MR/CT imaging applications^10^,^20^,^27^. On the other hand, star-shaped Au shells with strong SPR band in NIR region enable their applications in PA imaging and PTT of tumors *in vivo*^18^. In this work, we designed the Fe_3_O_4_ @ Au-PEI.Ac-PEG-FA NSs with targeting specificity to FAR-expressing cancer cells for multi-modal MR/CT/PA imaging and PTT of tumors *in vivo*. Compared to our previous study utilizing hyaluronic acid as a targeting ligand^39^, our current work aimed to expand the scope of the designed platform for theranostics of different cancer phenotypes.

Through depositing Au shell on the surface of Fe_3_O_4_/Ag seeds (Supplementary Fig. S1), the Fe_3_O_4_ @ Au core/shell NSs were formed and then successively decorated with PEI (4.8%) *via* the formation of Au-S bond and COOH-PEG-FA (7.6%) *via* the formation of amide bond (Supplementary Fig. S4). After acetylation of the remaining PEI surface amines (Supplementary Table S1), multifunctional Fe_3_O_4_ @ Au-PEI.Ac-PEG-FA NSs were formed. The formed NSs have a slightly positive surface potential (+14.4 mV), which doesn’t seem to exert any appreciable *in vivo* toxicity. This can be confirmed by monitoring the physiological status of mice after IV treatment of NSs for at least 10 days, in agreement with our previous work^54^. It should be mentioned that the designed Fe_3_O_4_ @ Au-PEI.Ac-PEG-FA NSs have a low molar ratio of Fe/Au (1:53.4). Due to the fact that we prepared the Fe_3_O_4_ seed NPs using a mild reduction route, the formed Fe_3_O_4_ @ Au core/shell NSs have a r_2_ relaxivity (549.07 mM^−1^s^−1^) 3.8 times higher than the Fe_3_O_4_ @ Au core/shell NSs (144.39 mM^−1^s^−1^) reported in our previous work^39^. With the higher Au content than other Fe_3_O_4_ @ Au NPs^27^,^55^ and non-compromised MR imaging sensitivity, the designed NSs should be able to used for sensitive MR and CT imaging. Our results reveal that the NSs display good MR relaxometry, X-ray attenuation, PA property and photothermal performance (Fig. 2), which is due to the co-existence of Fe_3_O_4_ core and star-shaped Au shell. The η of the NSs (88.9%, Supplementary Fig. S7) is much higher than that of other major PTT agents reported in the literature^43^,^44^,^45^,^46^, which is likely ascribed to the particle’s volume, structure, and shape^56^. The FA modification of the particles rendered the NSs with specific affinity to FAR-overexpressing cancer cells *in vitro* (Fig. 3a–g) and the xenografted tumor model *in vivo* (Fig. 4b,d and Supplementary Fig. S11). Hence the NSs hold great promise to be used as a nanoprobe for specific MR/CT/PA imaging of a FAR-expressing tumor model *in vivo* after either IT or IV injection (Figs 4 and 5). The tumor MR/CT/PA signal intensities after IV injection of the NSs are poorer than those of the corresponding imaging signals after IT injection of the NSs. This should be due to the fact that much less NSs were accumulated in tumors *via* IV injection than *via* IT injection. Additionally, the NIR-absorption feature of NSs affords their uses for efficient PTT of cancer cells *in vitro* and the tumor model *in vivo* (Figs 3h and 6). It should be mentioned that due to the fact that NPs with a size of 50–300 nm regardless of surface modification of targeting ligands have a significant accumulation in the tumor region after IT injection^33^,^44^,^57^, we used IT injection route to evaluate the PTT efficacy of the tumors *in vivo* using the designed NSs.

To conclude, we presented a novel route to generating Fe_3_O_4_ @ Au-PEI.Ac-PEG-FA NSs that can be used as a unique platform for multi-mode MR/CT/PA imaging and PTT of tumors. *Via* the integration of mild reduction route, seed-mediated growth method, and PEI-mediated conjugation, multifunctional Fe_3_O_4_ @ Au-PEI.Ac-PEG-FA NSs are able to be formed. The designed NSs possess excellent colloidal stability, good hemocompatibility/cytocompatibility, ultrahigh r_2_ relaxivity, good X-ray attenuation and PA property, and strong NIR absorption feature. These properties afford their uses as a theranostic nanoprobe for multi-mode imaging-guided PTT of tumors. Furthermore, the synthesized FA-modified Fe_3_O_4_ @ Au core/shell NSs are likely to be used for theranostics of different types of cancer for further translational medicine applications.

## Methods

### Materials

All chemicals and materials were from commercial resources and used as received. Details can be seen in Supplementary Information.

### Characterization techniques

The intermediate products and Fe_3_O_4_ @ Au-PEI.Ac-PEG-FA NSs were thoroughly characterized *via* hydrodynamic size and zeta potential measurements, ^1^H NMR spectroscopy, thermal gravimetric analysis (TGA), UV-vis spectroscopy, transmission electron microscopy (TEM), Leeman Prodigy inductively coupled plasma-optical emission spectroscopy (ICP-OES), T_2_ relaxometry measurements, MR/CT/PA phantom studies, and photothermal performance.

### Preparation of Fe_3_O_4_ @ Au NSs

Fe_3_O_4_/Ag seed particles were prepared by a protocol adopted from the literature^10^,^40^,^41^,^58^. To grow Au NSs onto the surface of Fe_3_O_4_ NPs, an Au growth solution with three different proportions of regents (AgNO_3_, AA, and HAuCl_4_) was used. Only the mixture that changed to blue and exhibited strong NIR-absorption in the UV-vis-NIR spectrum was regarded as the best option of Au growth solution for the formation of Fe_3_O_4_ @ Au NSs.

### Synthesis of Fe_3_O_4_ @ Au-PEI.Ac-PEG-FA NSs

Firstly, PEI-SH and COOH-PEG-FA were synthesized using protocols illustrated in the literature^19^,^39^,^49^,^59^. Thereafter, the above Fe_3_O_4_ @ Au NSs were successively modified with PEI-SH *via* the formation of Au-S bond and COOH-PEG-FA *via* the formation amido linkage. After neutralizing the remaining PEI amines, the Fe_3_O_4_ @ Au-PEI.Ac-PEG-FA NSs were obtained.

### Hemolysis and cytotoxicity assay

Hemolysis assay was performed by exposing human red blood cells (HRBCs) to a phosphate buffered saline (PBS) solution containing Fe_3_O_4_ @ Au-PEI.Ac-PEG-FA NSs in the Au concentration range of 0–4 mM for 2 h at room temperature. HeLa cells were routinely cultured and passaged in 25 cm^2^ plates at 37 °C and 5% CO_2_ in regular FA-free DMEM with 10% FBS, 1% penicillin/streptomycin. HeLa cells cultured in FA-free medium expressed high-level FAR (denoted as HeLa-HFAR cells)^60^,^61^, while HeLa cells grown in the DMEM containing 2.5 μM free FA for 24 h or longer expressed low-level FAR (denoted as HeLa-LFAR). Without specific statement, the term of “HeLa cells” is always deemed to be “HeLa-HFAR cells”. Cytotoxicity assay was conducted by culturing HeLa cells with fresh DMEM containing Fe_3_O_4_ @ Au-PEI.Ac-PEG-FA NSs in the tested Au concentration range (0–2 mM) for 24 h. MTT assay and cell morphology observation were used to quantitatively and qualitatively assess the cell viability.

### *In vitro* specific cellular uptake assay

After incubation of both HeLa-HFAR and HeLa-LFAR with Fe_3_O_4_ @ Au-PEI.Ac-PEG-FA NSs for 4–6 h, ICP-OES, TEM, MR imaging, and CT imaging were performed to determine or demonstrate the specific uptake of the NSs by HeLa-HFAR cells.

### *In vitro* photothermal ablation of HeLa cells

After cultivation with DMEM containing PBS (control) or Fe_3_O_4_ @ Au-PEI.Ac-PEG-FA NSs in the studied Au concentration range for 6 h, adherent HeLa cells were then rinsed with PBS and subjected to laser irradiation. MTT viability assay and cell morphology observation were then performed to quantitatively and qualitatively assess the cell viability, respectively.

### *In vivo* MR/CT/PA tri-modal imaging of a xenografted tumor model

All animal experiments were conducted in compliance with institutional guidelines and the policy of the National Ministry of Health, and were approved by the Institutional Animal Care and Use Committee of Zhongshan Hospital, Fudan University. A PBS solution (0.1 mL) containing the Fe_3_O_4_ @ Au-PEI.Ac-PEG-FA NSs ([Fe] = 1.31 mM) was delivered into each tumor-bearing mouse *via* an intravenous (IV) or intratumoral (IT) injection route. For MR imaging, tumor 2D MR images were collected before and at 0.5 h post IT injection or at 6 h post IV injection of the NSs by using a Signa HDxt superconductor clinical MR system (1.5 T). To verify the targeting specificity of NSs to FAR-expressing HeLa tumor, IV injection of free FA was first performed to block the FAR expression, followed by IV injection of the NSs. Tumor CT and PA imaging were further carried out with a GE LightSpeed VCT imaging system and the Vevo LAZR PA Imaging System, respectively.

### *In vivo* photothermal ablation of HeLa tumors

Intravital thermal imaging of HeLa tumor-bearing mice was first conducted at different laser irradiation time periods using an infrared camera. For PTT of tumors, the mice were randomly allocated into four groups and subjected to different treatments: Control group (without any treatment), PBS + Laser group (PBS with laser), NSs group (NSs without laser), and NSs + Laser group (NSs with laser). Thereafter, the size of the tumors, body weight and tumor volume were recorded at different time points. More experimental details can be found in Supplementary information.

## Additional Information

**How to cite this article**: Hu, Y. *et al*. Multifunctional Fe_3_O_4_ @ Au core/shell nanostars: a unique platform for multimode imaging and photothermal therapy of tumors. *Sci. Rep.* **6**, 28325; doi: 10.1038/srep28325 (2016).

## Supplementary Material

Supplementary Information
