# Supplementary material for: Multifunctional Fe3O4 @ Au core/shell nanostars: a unique platform for multimode imaging and photothermal therapy of tumors
Source: Sci Rep. 2016 Jun 21;6:28325. doi: 10.1038/srep28325 (PMC4914846; doi:10.1038/srep28325)
Supplement: Supplementary Information [file srep28325-s1.doc]

Supplementary Information for

Multifunctional Fe_3_O_4_@Au core/shell nanostars: a unique platform for multimode imaging and photothermal therapy of tumors

Yong Hu,^1^ Ruizhi Wang,^2^ Shige Wang,^3^ Ling Ding,^1^ Jingchao Li,^1^ Yu Luo,^1^ Xiaolin Wang,^2^*Mingwu Shen,^1^* Xiangyang Shi^1^*

^1^ State Key Laboratory for Modification of Chemical Fibers and Polymer Materials, College of Chemistry, Chemical Engineering and Biotechnology, Donghua University, Shanghai 201620, People’s Republic of China.

^2^ Shanghai Institute of Medical Imaging, Department of Interventional Radiology, Zhongshan Hospital, Fudan University, Shanghai 200032, People’s Republic of China.

^3^ College of Science, University of Shanghai for Science & Technology, Shanghai 200093, People’s Republic of China.

______________________________________________

Correspondence and requests for materials should be addressed to X. S. (xshi@dhu.edu.cn) or M. S. ([mwshen@dhu.edu.cn](mailto:mwshen@dhu.edu.cn)) or X. W. (xlwang-shmu@vip.sina.com)

Part of the experimental details

**Materials:** Heterofunctional PEG with dual amine and carboxyl ends (NH_2_-PEG-COOH, Mw = 2000) was purchased from Shanghai Yanyi Biotechnology Corporation (Shanghai, China). Ferric chloride hexahydrate (FeCl_3_·6H_2_O > 99%), sodium sulfite, ammonia (25-28%), CA, AgNO_3_, ascorbic acid (AA), and HAuCl_4_·4H_2_O were from Sinopharm Chemical Reagent Co., Ltd (Shanghai, China). NaBH_4_, branched PEI (Mw = 25000), dimethyl sulfoxide (DMSO), triethylamine, and acetic anhydride were supplied by Aldrich (St. Louis, MO). FA, 1-ethyl-3-[3-dimethylaminopropyl] carbodiimide hydrochloride (EDC), N-hydroxysuccinimide (NHS), cetyltrimethyl-ammoniumbromide (CTAB), and methyl thioglycolate (MTG) were supplied by J&K Chemical Ltd (Shanghai, China). HeLa cells (a human cervical cell line) were from Institute of Biochemistry and Cell Biology, the Chinese Academy of Sciences (Shanghai, China). Dulbecco's modified Eagle medium (DMEM), penicillin, streptomycin, and fetal bovine serum (FBS) were acquired from Hangzhou Jinuo Biomedical Technology (Hangzhou, China). 3-(4,5-Dimethylthiazol-2-yl)-2,5-diphenyltetrazolium bromide (MTT) was purchased from Shanghai Sangon Biological Engineering Technology & Services Co., Ltd. (Shanghai, China). All chemicals and reagents were used as received. Water used in all experiments was purified by a Milli-Q Plus 185 water purification system (Millipore, Bedford, MA) to have a resistivity higher than 18.2 MΩ·cm.

**Synthesis of Fe_3_O_4_/Ag seeds:** Fe_3_O_4_/Ag seeds were synthesized by a protocol inspired by the previous work[^1-4^](#_ENREF_1). Firstly, CA-stabilized Fe_3_O_4_ NPs were synthesized *via* a mild reduction route according to our previous work[^1^](#_ENREF_1). In brief, FeCl_3_·6H_2_O (1.3 g) dissolved in water (20 mL) was placed in a three-necked flask with a volume of 250 mL. After nitrogen bubbling and magnetic stirring for 15 min, a fresh aqueous solution of sodium sulfite (0.2 g, 10 mL) was added into the above solution under stirring. After 30 min, CA (0.5 g, in 5 mL water) and ammonia (2 mL) were sequentially added into the above mixture solution under stirring. The mixture solution was continuously stirred for another 30 min at 60-70 °C. Thereafter, the reaction mixture was cooled down to room temperature and stirred for an additional 1.5 h. The thus formed Fe_3_O_4_@CA product was collected with magnet and washed with water for several times. Finally, the Fe_3_O_4_@CA product was centrifuged (8000 rpm, 10 min) to remove the aggregated particles and the supernatant suspension was collected. Then, AgNO_3_ (3.4 mg, in 1 mL water) was added into the aqueous suspension of the Fe_3_O_4_@CA NPs (1 mg/mL, 10 mL) under vigorous magnetic stirring. After 15 min, an icy cold NaBH_4_ solution (3.8 mg, 1.8 mL in water-methanol, *v/v* = 2 : 1) was rapidly dropped into the above mixture and the mixture was continuously stirred for 2 h to form the Fe_3_O_4_@Ag CNPs. The obtained CNPs were magnetically collected and rinsed with water for several times, finally redispersed in water (10 mL) for further use.

**Synthesis of Fe_3_O_4_@Au NSs:** To grow Au NSs onto the the surface of Fe_3_O_4_ NPs, an Au growth solution (15 mL) with three different formulas was used, namely, mixture of 100 mM CTAB and (a) 0.08 mM AgNO_3_, 5.2 mM AA, and 1.6, 2.4, or 3.2 mM HAuCl_4_; (b) 0.08 mM AgNO_3_, 2.4 mM HAuCl_4_, and 4.0, 5.2, or 8.0 mM AA; or (c) 5.2 mM AA, 2.4 mM HAuCl_4_, and 0.04, 0.08, or 0.80 mM AgNO_3_. Then Fe_3_O_4_/Ag seeds (220 μg, 220 μL) were respectively dropped into the above mentioned mixtures and the color of each mixture gradually became deep within several minutes. Only the mixture that changed to blue and exhibited strong NIR-absorption in the UV-vis-NIR spectrum was regarded as the best choice of Au growth solution for the formation of Fe_3_O_4_@Au NSs. After being stirred for another 2 h, the product was centrifuged (6000 rpm, 5 min) and redispersed in water for three cycles to remove CTAB, and finally redispersed in 10 mL water for further use.

**Formation of Fe_3_O_4_@Au-PEI.Ac-PEG-FA NSs:** PEI-SH was first synthesized using a protocol illustrated in the literature. Thereafter, 10 mL of the above Fe_3_O_4_@Au NSs was mixed with 10 mL of PEI-SH (0.1 g) in aqueous solution, followed by sonication for 30 min and stirring for 24 h at room temperature. After that, the Fe_3_O_4_@Au-PEI NSs were purified *via* 3 cycles of centrifugation (6000 rpm, 5 min)/redispersion in water, and finally redispersed in DMSO (10 mL) before further use.

According to our previous work, COOH-PEG-FA (1.13 mg) was synthesized and then activated by EDC (0.89 mg) and NHS (0.53 mg) in 5 mL of DMSO under vigorous magnetic stirring for 3 h. Then, the activated COOH-PEG-FA was added into 10 mL of the above DMSO solution of the Fe_3_O_4_@Au-PEI NSs under stirring. Three days later, the raw product of Fe_3_O_4_@Au-PEI-PEG-FA NSs was subjected to 3 cycles of centrifugation/redispersion in water and acetylation to neutralize the remaining PEI amines according to the literature[^9^](#_ENREF_9). Briefly, triethylamine (12.6 μL) was added to the above solution containing Fe_3_O_4_@Au-PEI-PEG-FA NSs. After 30 min, acetic anhydride (10.3 μL) was dropped to the NSs/triethylamine mixture solution under stirring for an additional 24 h. Finally, the product was collected and purified *via* 3 cycles of centrifugation (6000 rpm, 5 min)/redispersion in water to get the Fe_3_O_4_@Au-PEI.Ac-PEG-FA NSs. The particles were stored in water or phosphate buffered saline (PBS) for further use.

**Characterization techniques:** Hydrodynamic size and zeta potential measurements were performed using a Malvern Zetasizer Nano ZS model ZEN3600 (Worcestershire, UK) equipped with a standard 633 nm laser. A Bruker AV400 nuclear magnetic resonance spectrometer was used to record the ^1^H NMR spectra of COOH-PEG-FA and PEI-SH specimens dissolved in D_2_O. Thermal gravimetric analysis (TGA) was performed using a TG 209 F1 (NETZSCH Instruments Co., Ltd., Selb/Bavaria, Germany) thermal gravimetric analyzer. The samples were heated from ambient temperature to 700 ^o^C under N_2_ atmosphere at a rate of 10 ^o^C/min. UV-vis spectroscopy of the specimens dispersed in water was conducted using a Lambda 25 UV-vis spectrophotometer (PerkinElmer, Boston, MA). Transmission electron microscopy (TEM, JEOL 2010F, Tokyo, Japan) was operated at an accelerating voltage of 200 kV. Prior to measurements, each specimen was prepared by dropping a particle suspension in water (5 μL, 0.1 mg/mL) onto a carbon-coated copper grid, where it was undisturbed until air dried. Leeman Prodigy inductively coupled plasma-optical emission spectroscopy (ICP-OES, Hudson, NH) was carried out to determine the Fe and Au concentrations of the samples dispersed in water or PBS. T_2_ relaxometry measurements and T_2_-weighted MR phantom studies were carried out using an NMI20-Analyst NMR Analyzing and Imaging system (Shanghai Niumag Corporation, Shanghai, China). The specimens were dispersed in water at different Fe concentrations (0.00125-0.02 mM) before measurements. The parameters were set as follows: CPMG sequence, 0.5 T magnet, TR = 6000 ms, TE = 80 ms, number of excitation = 1, point resolution = 156 mm × 156 mm, and section thickness = 0.6 mm. T_2_ relaxivity (r_2_) was calculated by fitting the inverse T_2_ relaxation time (1/T_2_) as a function of the Fe concentration. CT imaging was performed using a GE LightSpeed VCT imaging system (GE Medical Systems, Milwaukee, WI) with 120 kV, 100 mA, and 0.625 mm slice thickness. The specimens were dispersed in water at different Au concentrations (4-64 mM) before measurements. For PA imaging, individual hole on a home-made agar plate with different Au concentrations (0.25, 0.5, 1.0, and 2.0 mM, respectively) was monitored using the Vevo LAZR PA Imaging System (VisualSonics Inc., Toronto, Canada) under an 808 nm laser (~6 mJ/cm^2^). A series of specimens with different Au concentrations (1, 2, 4, 10, and 20 mM, respectively) were put into quartz cuvettes, respectively, and then irradiated by an 808 nm laser (facula area: 0.3 cm^2^, output power density: 1.0 W/cm^2^) using a laser device (Shanghai Xilong Optoelectronics Technology Co. Ltd., Shanghai, China) for 300 s to determine the concentration-based photothermal property. In addition, a series of specimens with the same Au concentration (10 mM, 0.1 mL) were put into quartz cuvettes, respectively, and irradiated by an 808 nm laser (as mentioned above) for 300 s with different output power densities (0.25, 0.5, 1.0, and 1.5 W/cm^2^, respectively) to further determine the laser energy-based photothermal property. An in situ DT-8891E thermocouple thermometer (Shenzhen Everbest Machinery Industry Co., Ltd., Shenzhen, China) was used to record the temperature change of different samples every 5 s. Furthermore, by monitoring a thermocouple, the temperature change of the Fe_3_O_4_@Au-PEI.Ac-PEG-FA NSs in aqueous solution (83.1 μg/mL, 0.1 mL, OD_808_ = 1.98) irradiated with a 808 nm laser under an output power density of 1.0 W/cm^2^ was specifically investigated during both heating (laser on) and cooling (laser off) stages, followed by calculation of the photothermal conversion efficiency (η).

**Hemolysis and cytotoxicity assay:** Fresh human blood stabilized with heparin was kindly supplied by Shanghai Zhongshan Hospital (Shanghai, China) with the approval of the Shanghai Zhongshan Hospital Ethical Committee. The human red blood cells (HRBCs) were obtained according to the previous reported protocol[^10^](#_ENREF_10). Hemolysis assay was performed according to the literature[^11^](#_ENREF_11). In brief, 0.1 mL of 10-fold diluted HRBC suspension was gently mixed with 0.9 mL of PBS containing Fe_3_O_4_@Au-PEI.Ac-PEG-FA NSs at different Au concentrations (0.5, 1.0, 2.0, and 4.0 mM, respectively). The mixtures were maintained still for 2 h at room temperature, centrifuged (10000 rpm, 1 min), and then the photographs of the samples were taken while a PerkinElmer Lambda 25 UV-vis spectrophotometer was used to measure the absorbance of the hemoglobin. PBS and water were separately used as a negative and a positive control. The hemolysis percentages of different samples were calculated at 541 nm according to a previously reported method[^12^](#_ENREF_12).

*In vitro* cytotoxicity of the Fe_3_O_4_@Au-PEI.Ac-PEG-FA NSs was evaluated by MTT viability assay of HeLa cells according to the literature[^13^](#_ENREF_13). Briefly, HeLa cells in 200 μL of fresh DMEM were seeded into a 96-well plate at an initial density of 1 × 10^4^ cells/well. After incubation overnight at 37 °C and 5% CO_2_ to bring the cells to confluence, the medium was replaced with 200 μL of fresh medium containing PBS (control) and Fe_3_O_4_@Au-PEI.Ac-PEG-FA NSs with different Au concentrations (0.2, 0.4, 0.8, 1.5, 2.0, 4.0 and 8.0 mM, respectively), and the cells were further incubated at 37 ^o^C and 5% CO_2_ for another 24 h. After that, MTT (20 μL, 5 mg/mL in PBS) was added to each well and the cells were incubated continually for an additional 4 h. Thereafter, 150 μL of DMSO was added to each well to replace the medium and to dissolve the insoluble formazan crystals. A Thermo Scientific Multiskan MK3 ELISA reader (Thermo Scientific, Waltham, MA) was used to record the absorbance at 570 nm in each well. Mean and standard deviation (SD) of 5 parallels were reported for each sample.

To further investigate the cytotoxicity of the Fe_3_O_4_@Au-PEI.Ac-PEG-FA NSs, the morphology of cells treated with PBS (control) and Fe_3_O_4_@Au-PEI.Ac-PEG-FA NSs at different Au concentrations (0.8, 1.5, 2.0, 4.0 and 8.0 mM, respectively) for 24 h was also observed using a Leica DM IL LED inverted phase contrast microscope with a 200 × magnification for each sample.

***In vitro* specific cellular uptake assay:** ICP-OES was performed to quantify the specific uptake of the Fe_3_O_4_@Au-PEI.Ac-PEG-FA NSs by HeLa-HFAR cells. Both HeLa-HFAR and HeLa-LFAR cells in 1 mL of DMEM were seeded in 12-well culture plates at a density of 2 × 10^5^ cells/well. After incubation overnight at 37 ^o^C and 5% CO_2_ to bring the cells to confluence, the medium was carefully discarded and replaced with 1 mL of fresh medium containing PBS (control) and Fe_3_O_4_@Au-PEI.Ac-PEG-FA NSs at different Au concentrations (0.5, 1.0, and 2.0 mM, respectively). After incubation for an additional 4 h, the cells were successively rinsed with PBS for 5 times, harvested (by trypsinization and centrifugation), and counted by Handheld Automated Cell Counter (Millipore corporation, Billerica, MA). The collected cells were lysed using an aqua regia solution (2.0 mL) for 2 days prior to ICP-OES assay.

The specific uptake of the Fe_3_O_4_@Au-PEI.Ac-PEG-FA NSs by HeLa-HFAR cells was further examined by TEM at a voltage of 60 kV. Briefly, HeLa-HFAR and HeLa-LFAR cells were seeded in 100-mm culture dish with 5 mL of fresh medium at a density of 1 × 10^6^ cells/mL. After incubation overnight at 37 ^o^C and 5% CO_2_ to bring the cells to confluence, the medium was substituted by fresh medium containing Fe_3_O_4_@Au-PEI.Ac-PEG-FA NSs with an Au concentration of 100 nM. After incubation for another 4 h, the medium was carefully removed and cells were washed with PBS for 3 times and fixed at 4 ^o^C for 1 h using glutaraldehyde (2.5% in PBS). Then the cells were further subjected to a sequence of treatments to obtain sections that were subsequently mounted onto copper grids before TEM measurements according to the literature.

The specific uptake of the Fe_3_O_4_@Au-PEI.Ac-PEG-FA NSs by HeLa-HFAR cells was further examined by cellular MR and CT imaging *in vitro*. HeLa-HFAR and HeLa-LFAR cells in 2 mL of fresh DMEM were separately seeded into 6-well culture plates at a density of 2 × 10^6^ cells/well and incubated at 37 ^o^C and 5% CO_2_ overnight. Then the medium was carefully removed and replaced with fresh medium containing PBS (control) and Fe_3_O_4_@Au-PEI.Ac-PEG-FA NSs at different Fe concentrations (0.0187, 0.0375, 0.075, and 0.15 mM, respectively) or Au concentrations (1, 2, 4, and 8 mM, respectively), and the cells were incubated at 37 °C and 5% CO_2_ for an additional 6 h. After that, the cells were rinsed 3 times with PBS, harvested (by trypsinization and centrifugation), and resuspended in 1 mL of PBS (containing 0.5% agarose) in 2-mL Eppendorf tubes prior to dual mode MR/CT imaging. A 1.5 T Signa HDxt superconductor clinical MR system (GE Medical Systems, Milwaukee, WI) was operated to obtain T_2_-weighted MR image of each specimen under the following parameters: TR = 4000 ms, TE = 71 ms, number of excitation = 1, point resolution = 156 mm × 156 mm, and section thickness = 0.6 mm. The cell specimens for CT imaging were prepared using protocols similar to those described above using Au concentration as a measure. A GE LightSpeed VCT imaging system (GE Medical Systems, Milwaukee, WI) was performed with 120 kV, 100 mA, and a slice thickness of 0.625 mm to collect CT image of each specimen.

***In vitro* photothermal ablation of HeLa cells:** HeLa cells in 200 μL DMEM were seeded into 96-well plates at a density of 1 × 10^4^ cells/well and incubated for 12 h to bring the cells to confluence. Then the medium was carefully discarded and replaced by 200 μL of fresh medium containing PBS (control) or Fe_3_O_4_@Au-PEI.Ac-PEG-FA NSs at different Au concentrations (0.1, 0.2, 0.4, and 0.8 mM, respectively). After incubation for another 6 h, the medium was replaced with fresh medium and continuously irradiated by an 808 nm laser with an output power density of 1.0 W/cm^2^ for 5 min. MTT viability assay (as described above) was then performed to quantitatively measure the cell viability.

Likewise, the cells were also treated with PBS or Fe_3_O_4_@Au-PEI.Ac-PEG-FA NSs in the Au concentration range of 0.1-0.8 mM for 6 h, followed by replacement with fresh medium and irradiation with an 808 nm laser at an output power density of 1.0 W/cm^2^ for 5 min. After being washed with PBS for 3 times, the cells were observed by Leica DM IL LED inverted phase contrast microscope with a 200 × magnification for each sample.

***In vivo* MR/CT/PA tri-modal imaging of a xenografted tumor model:** All animal experiments were conducted in compliance with institutional guidelines, and also in accordance with the policy of the National Ministry of Health. Male BALB/c nude mice (15-20 g) aged four to six weeks were purchased from Shanghai Slac Laboratory Animal Center (Shanghai, China). To establish the xenografted tumor model, 2 × 10^6^ HeLa cells/mouse were implanted subcutaneously into the right hind leg of the nude mice. Animal experiments were performed when the tumor average diameter reached 0.5-0.8 cm at approximately 4 weeks post implantation. The tumor-bearing mice were intraperitoneally anesthetized by injection of pentobarbital sodium (1.5% in normal saline, 40 mg/kg). Subsequently, a PBS solution containing the Fe_3_O_4_@Au-PEI.Ac-PEG-FA NSs was injected to the tumor-bearing mice *via* an intravenous (IV) or intratumoral (IT) injection route. For T_2_-weighted MR imaging of the tumor model, each mouse was imaged using a 1.5 T Signa HDxt superconductor clinical MR system under the same parameters set for *in vitro* imaging of cancer cells. After placing the mice inside an animal receiver coil (Chenguang Med Tech, Shanghai, China), two-dimensional (2D) spin-echo MR images were collected before and at 0.5 h post IT injection or at 6 h post IV injection of the Fe_3_O_4_@Au-PEI.Ac-PEG-FA NSs ([Fe] = 1.31 mM, 0.1 mL in PBS). In addition, *in vivo* free FA blocking experiment was designed to verify the targeting specificity of NSs to FAR-expressing HeLa tumor model. In brief, each mouse pre-treated with free FA (1 mM, 0.1 mL in PBS) for 1 h was injected with the Fe_3_O_4_@Au-PEI.Ac-PEG-FA NSs ([Fe] = 1.31 mM, 0.1 mL in PBS). MR images were collected before and at 6 h post IV injection of the NSs.

For CT imaging, a GE LightSpeed VCT imaging system was operated to image mice injected with the Fe_3_O_4_@Au-PEI.Ac-PEG-FA NSs ([Au] = 70 mM, in 0.1 mL PBS). The parameters were set in accordance with those used for *in vitro* CT imaging of cancer cells and the time points were the same as those used for *in vivo* MR imaging.

For PA imaging, the Vevo LAZR PA Imaging System with an 808 nm laser was performed to image tumor-bearing mice. Mice were treated by IT injection of the Fe_3_O_4_@Au-PEI.Ac-PEG-FA NSs ([Au] = 100 mM, in 0.04 mL PBS) and PA imaging was performed at 0.5 h postinjection. Mice were also treated by IV injection of the Fe_3_O_4_@Au-PEI.Ac-PEG-FA NSs ([Au] = 100 mM, 0.1 mL PBS) and the PA imaging was carried out at 6 h postinjection according to the literature[^16^](#_ENREF_16).

***In vivo* photothermal imaging:** After being anesthetized, HeLa tumor-bearing mice were intratumorally injected with PBS (control) or a PBS solution of the Fe_3_O_4_@Au-PEI.Ac-PEG-FA NSs ([Au] = 20 mM, 0.1 mL). At 0.5 h postinjection, the tumor sites were continuously exposed to an 808 nm laser with an output power density of 1.0 W/cm^2^ for 5 min. The dynamic whole-body infrared thermal images were collected by a photothermal medical device (FLIR A300, IRS Systems Inc., Shanghai, China) coupling with an infrared camera during laser radiation.

***In vivo* photothermal ablation of HeLa tumors:** HeLa tumor-bearing mice were randomly allocated into four groups and subjected to different treatments (n = 4 for each group). Namely, the mice were regularly fed without any treatment (Control group); the mice were intratumorally injected with PBS (0.1 mL) and then the tumor area was specifically exposed to an 808 nm laser with an output power density of 1.0 W/cm^2^ for 5 min (PBS + Laser group); and the mice were intratumorally injected with the Fe_3_O_4_@Au-PEI.Ac-PEG-FA NSs ([Au] = 20 mM, in 0.1 mL PBS) without laser irradiation (NSs group), and the Fe_3_O_4_@Au-PEI.Ac-PEG-FA NSs ([Au] = 20 mM, in 0.1 mL PBS) with laser irradiation under the same conditions as described above (NSs + Laser group). The repeated treatments were performed two and four days later (day 2 and day 4, respectively).

The body weight of all mice was measured using a counter balance, the size of the tumors was measured by using a digital vernier caliper, and pictures of mice were photographed at different time points. The tumor volume was calculated according to the equation of V = (tumor length × (tumor width)^2^)/2. The survival rate of the mice in each group was calculated according to the standard formula: N_1_/N × 100%, where N_1_ and N stand for the number of survival mice and total mice in each group, respectively.

**Histological examinations:** To further study the mechanism of *in vivo* tumor photothermal ablation and the effect of PTT at the level of histology, four groups of HeLa tumor-bearing mice were subjected to different treatments according to the procedures described above (n = 1 for each group). The mice were euthanized at 2 h posttreatment, and the tumors were extracted, fixed in 4% paraformaldehyde, and embedded in paraffin for Hematoxylin-Eosin (H&E) and TdT-mediated dUTP Nick-End Labeling (TUNEL) staining according to our previous work[^8^](#_ENREF_8). The morphology of tumor sections was randomly observed using a Leica DM IL LED inverted phase contrast microscope. The number of TUNEL-positive cells (apoptosis cells) in each specimen were counted and the percentages of apoptotic cells were calculated from five random fields of the images.

**Statistical analysis:** One-way ANOVA statistical analysis was used to assess the significance of the experimental data. A value of 0.05 was chosen as the significance level, and the data were marked as (*) for p < 0.05, (**) for p < 0.01, and (***) for p < 0.001, respectively.

**Calculation of photothermal conversion efficiency**

The photothermal conversion efficiency (η) of the NSs was calculated according to the literature using the following equation:

(1)

Where h, s, T_max_, T_sur_, I, A_808_, and Q_dis_ represent the heat transfer coefficient, the surface area of the container, the maximum equilibrium temperature, the surrounding temperature, the laser power, the absorbance (OD value) of the NSs at 808 nm, and a parameter generated by the incident laser, respectively.

Q_dis_ can be calculated according to Equation 2:

Q_dis_ = 10^3^·m·C·ΔT/t (2)

Where m, C, ΔT, and t represent the mass of water (g), the heat capacity of water (J g^-1^K^-1^), the elevation of temperature (K), and the laser irradiation time (s), respectively.

hs can be calculated according to Equation 3:

$\text{hs}=\text{ }\frac{\sum_{\text{i}}\text{m}_{\text{i}}\text{C}_{^{\text{p,i}}}}{\text{τ}_{\text{s}}}$ (3)

Where $\text{τ}_{\text{s}}$ and i stand for the time constant of the specimen system and the components of the system including NS suspension and specimen container.

The cooling time t and the driving force temperature θ can be calculated according to the following equations:

t = -$\text{τ}_{\text{s}}$lnθ (4)

θ = (T-T_sur_)/( T_max_-T_sur_) (5)

In this case, the temperature change of the NS solution (83.1 μg/mL, in 100 μL water, OD_808_ = 1.98) was recorded under the stages of heating by laser irradiation (808 nm, facula area: 0.3 cm^2^, output power density: 1.0 W/cm^2^) and subsequent cooling without the laser irradiation (laser off) (Supplementary Fig. S7a). The maximum temperature of the NS solution is increased by 34.3 ^o^C at 250 s postirradiation, which is ascribed to the thermal equilibrium between the heat input and output[^17^](#_ENREF_17). For comparison, according to the temperature curve of water (control) in Figure 2g, the temperature is only increased by 5.0 ^o^C. Q_dis_ was then calculated to be 8.4 mW using Equation (2). On the basis of the linear fitting the cooling time t *vs* the term of “-lnθ”, the time constant for heat transfer $\text{τ}_{\text{s}}$ (the slope of the plot) was determined to be 81 s (Fig. S7b). The “hs” was subsequently calculated to be 7.95 mW/^o^C using Equation (3). Ultimately, employing Equation (1), the photothermal conversion efficiency (η) of NSs was calculated to be 88.9%, which is prominently higher than that of other major PTT agents[^17-20^](#_ENREF_17).

**References:**

1 Hu, Y. *et al.* Facile synthesis of RGD peptide-modified iron oxide nanoparticles with ultrahigh relaxivity for targeted MR imaging of tumors. *Biomater. Sci.* **3**, 721-732 (2015).

2 Sanchez-Gaytan, B. L. & Park, S.-J. Spiky gold nanoshells. *Langmuir* **26**, 19170-19174 (2010).

3 Cheraghipour, E., Javadpour, S. & Mehdizadeh, A. R. Citrate capped superparamagnetic iron oxide nanoparticles used for hyperthermia therapy. *J. Biomed. Sci. Eng.* **5**, 715-719 (2012).

4 Bhana, S., Rai, B. K., Mishra, S. R., Wang, Y. & Huang, X. Synthesis and properties of near infrared-absorbing magnetic-optical nanopins. *Nanoscale* **4**, 4939-4942 (2012).

5 Li, J. *et al.* Hyaluronic acid-modified Fe_3_O_4_@Au core/shell nanostars for multimodal imaging and photothermal therapy of tumors. *Biomaterials* **38**, 10-21 (2015).

6 Pan, B. *et al.* Controlled self-assembly of thiol-terminated poly(amidoamine) dendrimer and gold nanoparticles. *Colloids Surf., A* **259**, 89-94 (2005).

7 Chen, Q. *et al.* Targeted CT/MR dual mode imaging of tumors using multifunctional dendrimer-entrapped gold nanoparticles. *Biomaterials* **34**, 5200-5209 (2013).

8 Zhu, J. *et al.* Targeted cancer theranostics using alpha-tocopheryl succinate-conjugated multifunctional dendrimer-entrapped gold nanoparticles. *Biomaterials* **35**, 7635-7646 (2014).

9 Zhou, B. *et al.* Synthesis and characterization of PEGylated polyethylenimine-entrapped gold nanoparticles for blood pool and tumor CT imaging. *ACS Appl. Mater. Interfaces* **6**, 17190-17199 (2014).

10 Shen, M. *et al.* Facile one-pot preparation, surface functionalization, and toxicity assay of APTS-coated iron oxide nanoparticles. *Nanotechnology* **23** (2012).

11 Li, J. *et al.* Hyaluronic acid-modified hydrothermally synthesized iron oxide nanoparticles for targeted tumor mr imaging. *Biomaterials* **35**, 3666-3677 (2014).

12 Zhao, Y., Wang, S., Guo, Q., Shen, M. & Shi, X. Hemocompatibility of electrospun halloysite nanotube- and carbon nanotube-doped composite poly(lactic-co-glycolic acid) nanofibers. *J. Appl. Polym. Sci.* **127**, 4825-4832 (2013).

13 Liu, H. *et al.* Facile formation of folic acid-modified dendrimer-stabilized gold-silver alloy nanoparticles for potential cellular computed tomography imaging applications. *Analyst* **138**, 1979-1987 (2013).

14 Shi, X. *et al.* Dendrimer-entrapped gold nanoparticles as a platform for cancer-cell targeting and imaging. *Small* **3**, 1245-1252 (2007).

15 Wang, S. *et al.* Dendrimer-functionalized iron oxide nanoparticles for specific targeting and imaging of cancer cells. *Adv. Funct. Mater.* **17**, 3043-3050 (2007).

16 Wang, S. *et al.* A facile one-pot synthesis of a two-dimensional MoS_2_/Bi_2_S_3_ composite theranostic nanosystem for multi-modality tumor imaging and therapy. *Adv. Mater.* **27**, 2775-2782 (2015).

17 Bhana, S. *et al.* Near-infrared-absorbing gold nanopopcorns with iron oxide cluster core for magnetically amplified photothermal and photodynamic cancer therapy. *ACS Appl. Mater. Interfaces* **7**, 11637-11647 (2015).

18 Tian, Q. *et al.* Hydrophilic Cu_9_S_5_ nanocrystals: A photothermal agent with a 25.7% heat conversion efficiency for photothermal ablation of cancer cells in vivo. *ACS Nano* **5**, 9761-9771 (2011).

19 Cole, J. R., Mirin, N. A., Knight, M. W., Goodrich, G. P. & Halas, N. J. Photothermal efficiencies of nanoshells and nanorods for clinical therapeutic applications. *J. Phys. Chem. C* **113**, 12090-12094 (2009).

20 Huang, P. *et al.* Biodegradable gold nanovesicles with an ultrastrong plasmonic coupling effect for photoacoustic imaging and photothermal therapy. *Angew. Chem., Int. Ed.* **52**, 13958-13964 (2013).


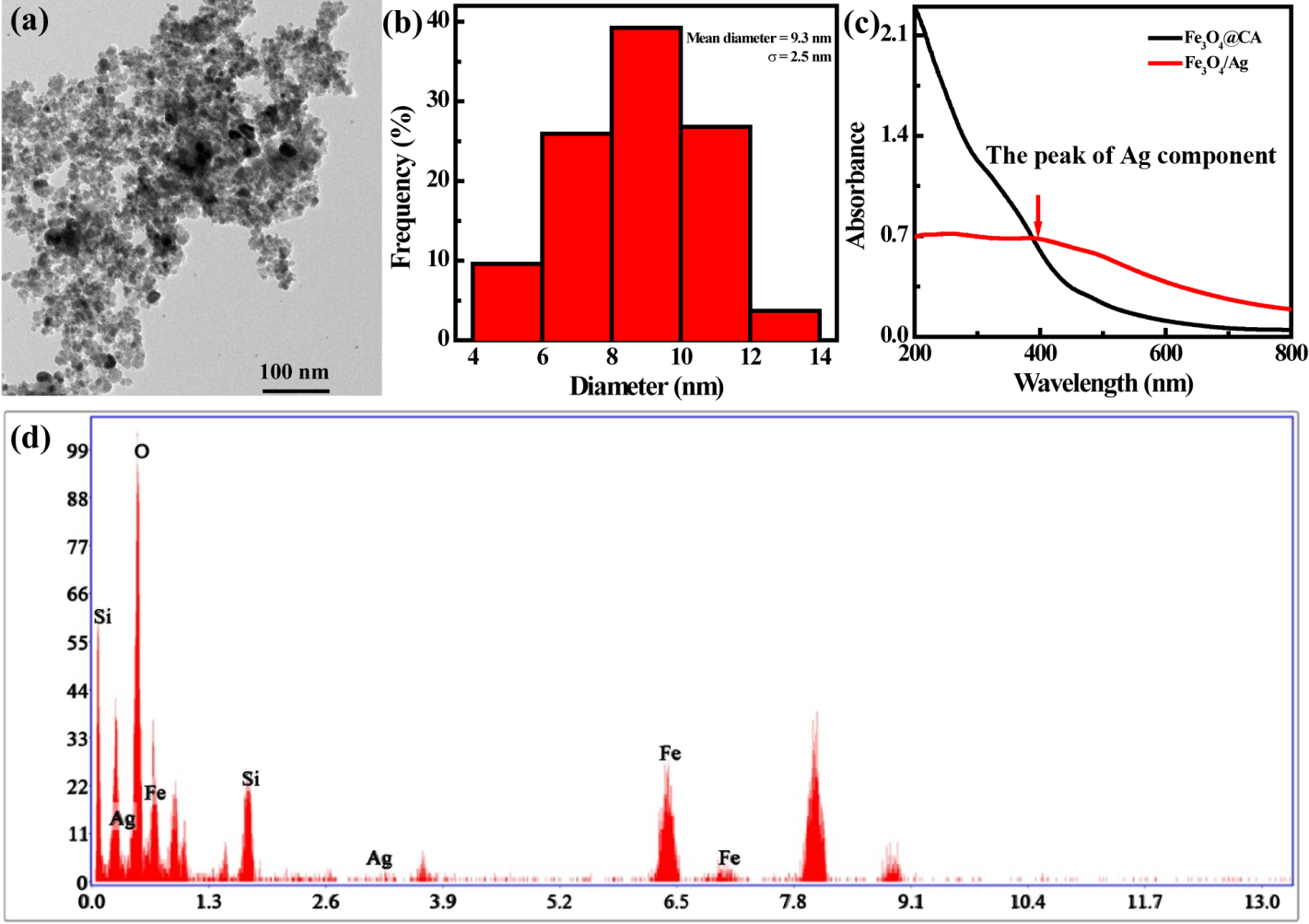


**Figure S1.** (a) TEM image, (b) size distribution histogram, (c) UV-vis spectrum, and (d) energy dispersive spectrum of the Fe_3_O_4_/Ag seeds. In (c), the UV-vis spectrum of Fe_3_O_4_@CA is also shown.


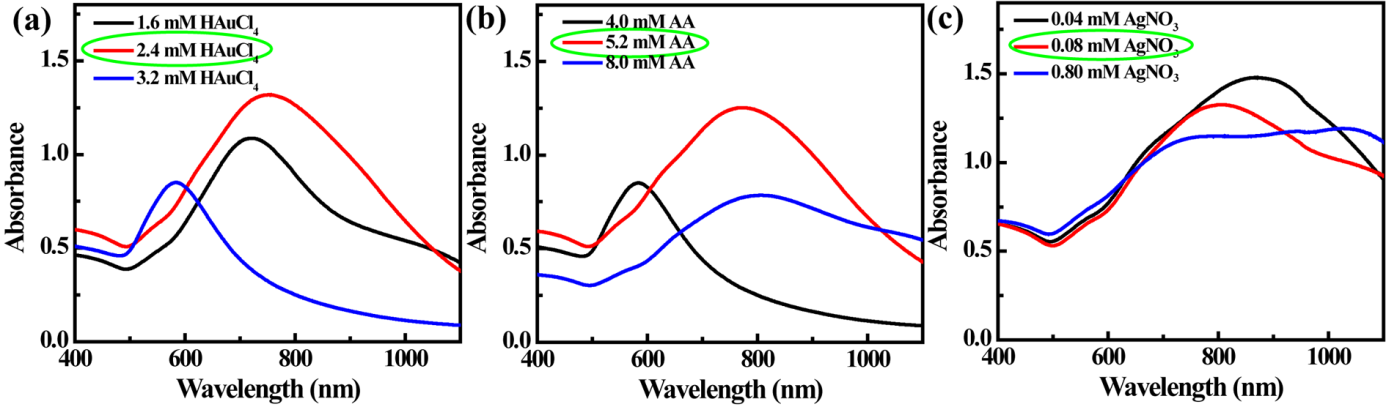


**Figure S2.** UV-vis spectra of the Fe_3_O_4_@Au NSs stabilized with CTAB grown in 15 mL growth solution containing different concentrations of HAuCl_4_, AA and AgNO_3_: (a) 0.08 mM AgNO_3_, 5.2 mM AA, and 1.6, 2.4, or 3.2 mM HAuCl_4_; (b) 0.08 mM AgNO_3_, 2.4 mM HAuCl_4_, and 4.0, 5.2, or 8.0 mM AA; (c) 5.2 mM AA, 2.4 mM HAuCl_4_, and 0.04, 0.08, or 0.80 mM AgNO_3_.

**
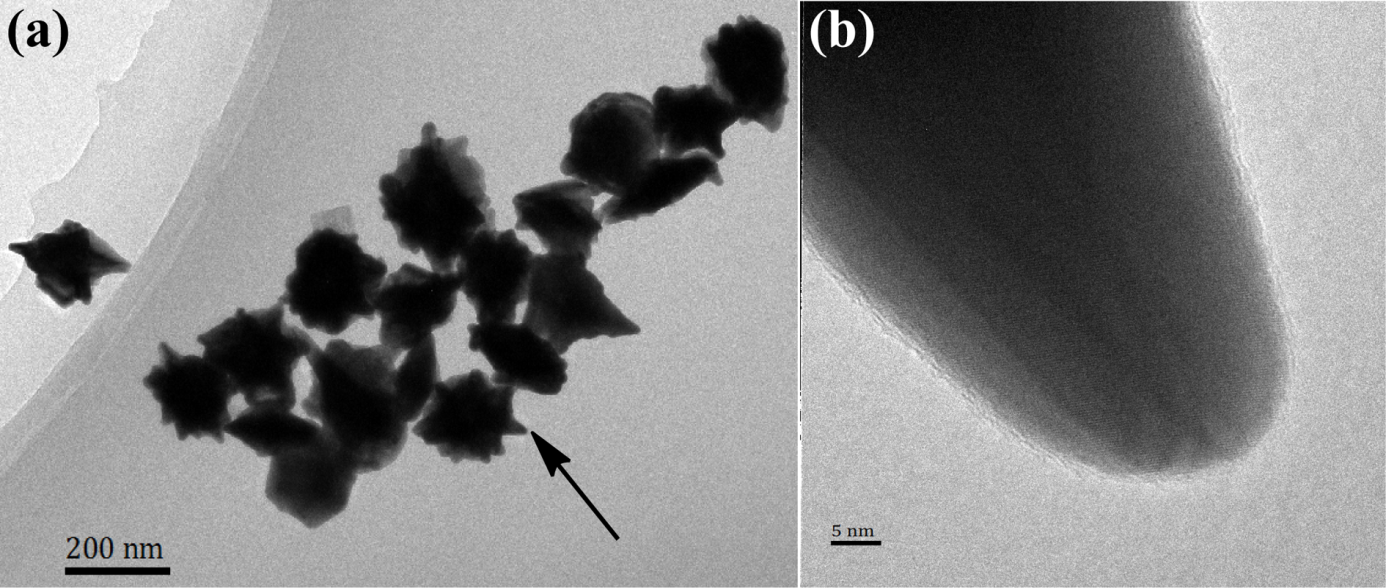
**

**Figure S3.** (a) TEM image of Fe_3_O_4_@Au NSs stabilized with CTAB. (b) shows a magnified TEM image of a single Au spike shown in (a) as indicated by the arrow.


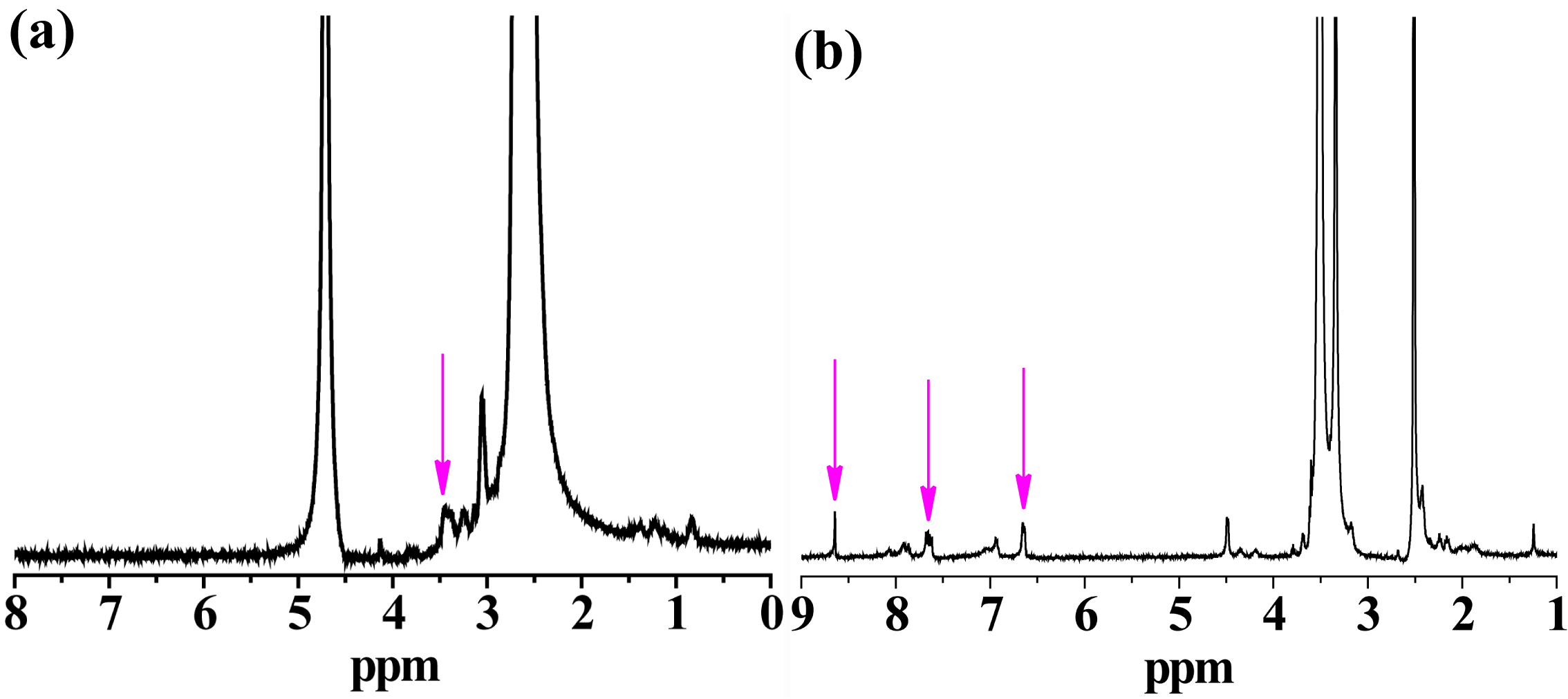


**Figure S4.** ^1^H NMR spectra of (a) PEI-SH (the peak at 3.4 ppm attributed to the proton signal of -CO-CH_2_-S- in MTG) and (b) COOH-PEG-FA (the peak at 6.62, 7.70, and 8.64 ppm attributed to the aromatic protons associated with FA) dissolved in D_2_O.


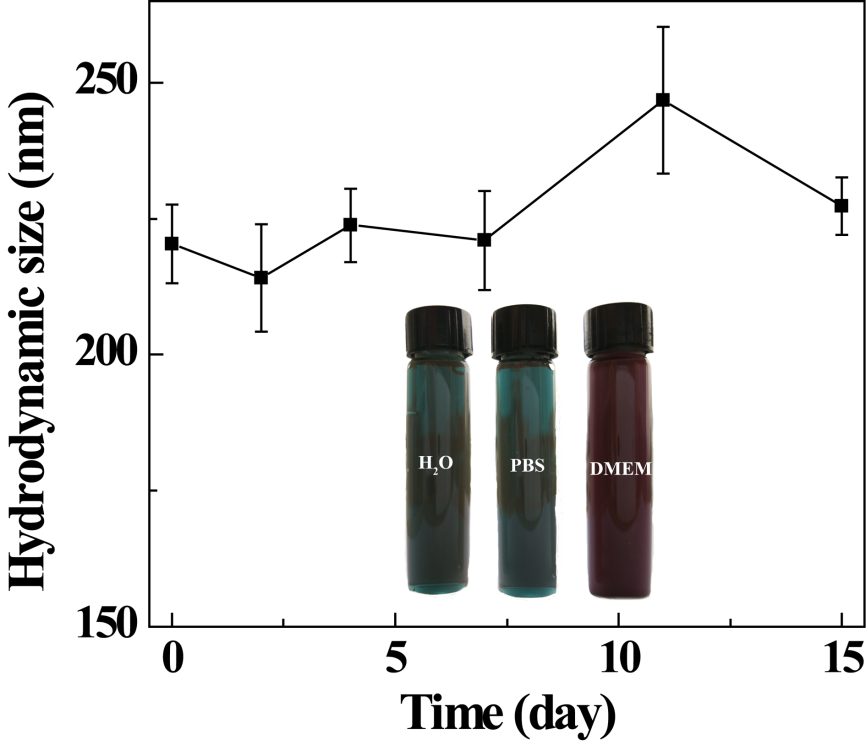


**Figure S5.** Hydrodynamic size of the Fe_3_O_4_@Au-PEI.Ac-PEG-FA NSs in different storage time periods. Inset shows the photographs of the Fe_3_O_4_@Au-PEI.Ac-PEG-FA NSs dispersed in water, PBS, and DMEM over one month, respectively.

**Figure S6.** TGA curves of the Fe_3_O_4_@Au, Fe_3_O_4_@Au-PEI, and Fe_3_O_4_@Au-PEG-FA NSs, respectively.


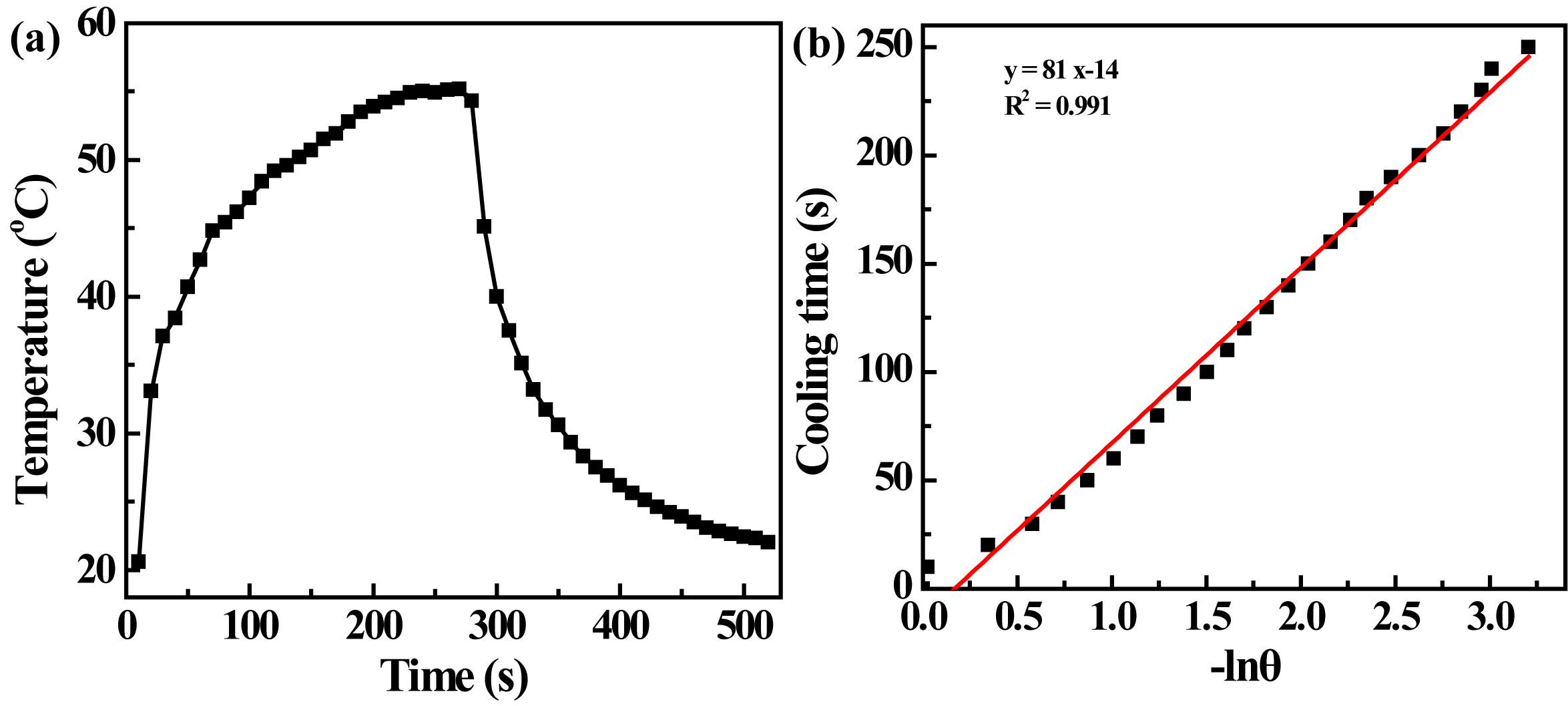


**Figure S7.** (a) Plot of the temperature *vs* time for the Fe_3_O_4_@Au-PEI.Ac-PEG-FA NSs (83.1 μg/mL, OD_808_ = 1.98) during laser irradiation (808 nm, 1.0 W/cm^2^) and cooling (laser off) stages. (b) Plot of the cooling time *vs* -lnθ. On the basis of the linear fitting analysis, the time constant for heat transfer $\text{τ}_{\text{s}}$ (the slope of the plot) was determined to be 81 s.

**
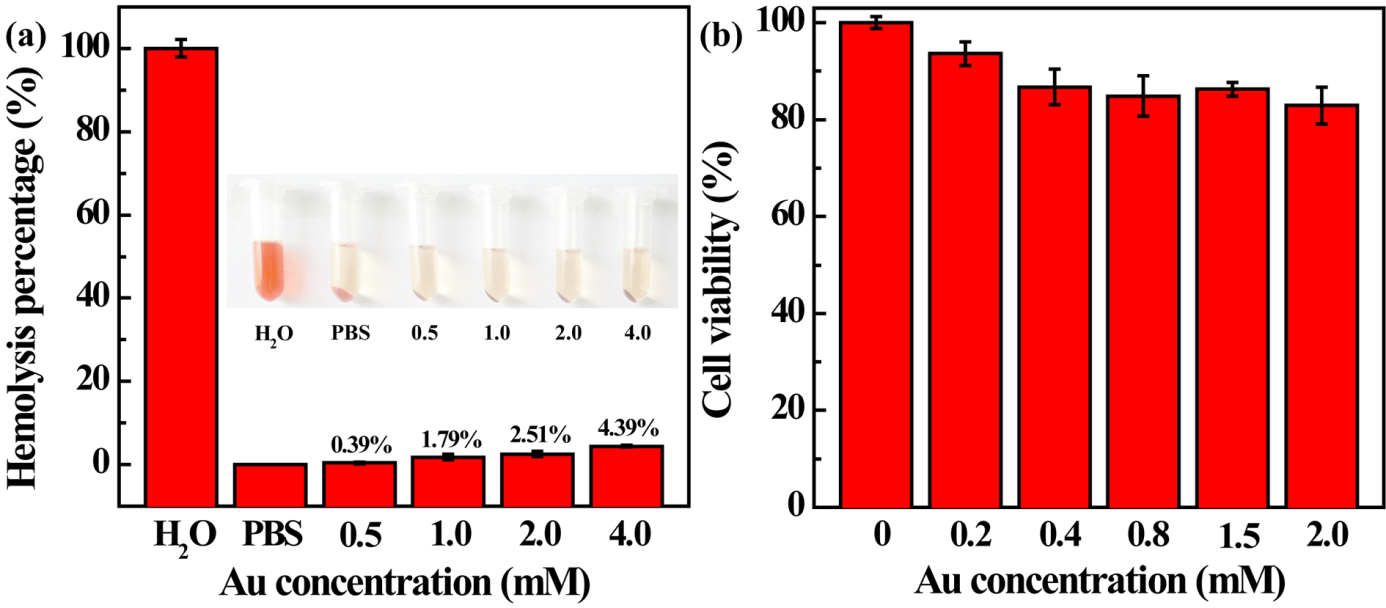
**

**Figure S8.** (a) Hemolytical activity of the Fe_3_O_4_@Au-PEI.Ac-PEG-FA NSs at different Au concentrations (0.5, 1.0, 2.0, and 4.0 mM, respectively). PBS and water were used as negative and positive controls, respectively. The inset shows the photograph of HRBCs exposed to water, PBS, and PBS containing NSs at different Au concentrations for 2 h followed by centrifugation. (b) MTT assay of HeLa cell viability after treatment with the Fe_3_O_4_@Au-PEI.Ac-PEG-FA NSs in the Au concentration range of 0-2.0 mM for 24 h.


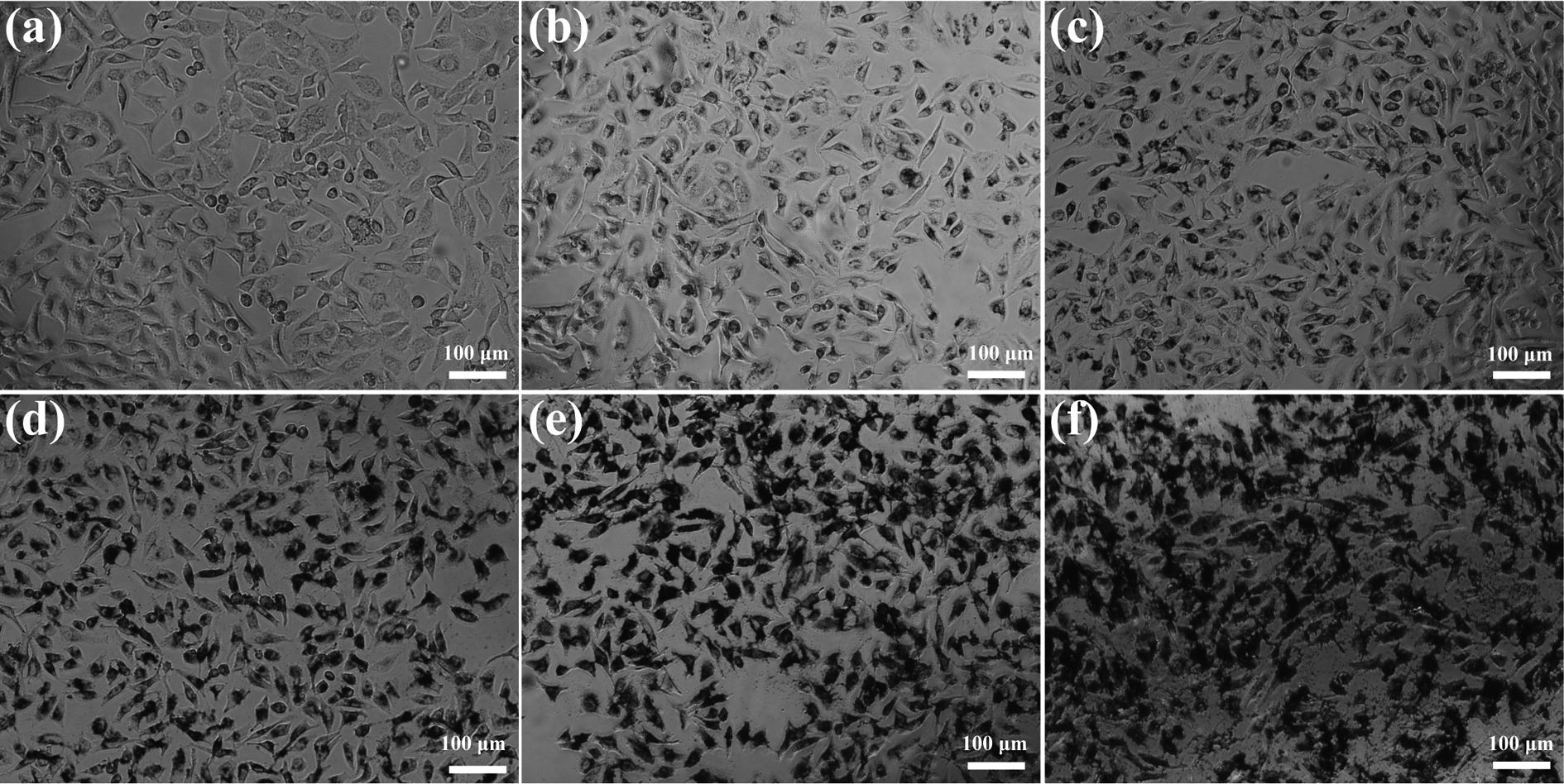


**Figure S9.** Phase contrast microscopic images of HeLa cells treated with (a) PBS and Fe_3_O_4_@Au-PEI.Ac-PEG-FA NSs at the Au concentrations of (b) 0.2 mM, (c) 0.4 mM, (d) 0.8 mM, (e) 1.5 mM, and (f) 2.0 mM for 24 h. The scale bar in each panel represents 100 μm.

**
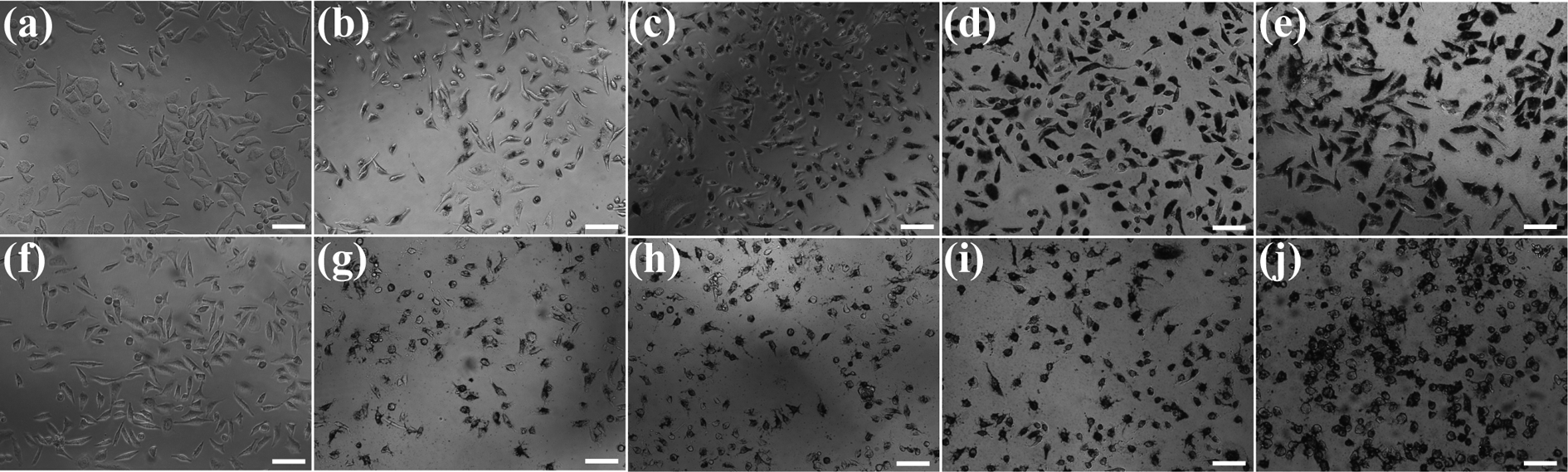
**

**Figure S10.** Phase contrast microscopic images of HeLa cells treated with (a) PBS, HeLa cells treated with the Fe_3_O_4_@Au-PEI.Ac-PEG-FA NSs alone at the Au concentrations of (b) 0.1 mM, (c) 0.2 mM, (d) 0.4 mM, and (e) 0.8 mM for 6 h, and HeLa cells first treated with (f) PBS or the Fe_3_O_4_@Au-PEI.Ac-PEG-FA NSs at the Au concentrations of (g) 0.1 mM, (h) 0.2 mM, (i) 0.4 mM, (j) 0.8 mM for 6 h and then irradiated by an 808 nm laser for 5 min. The scale bar in each panel represents 100 μm.

**
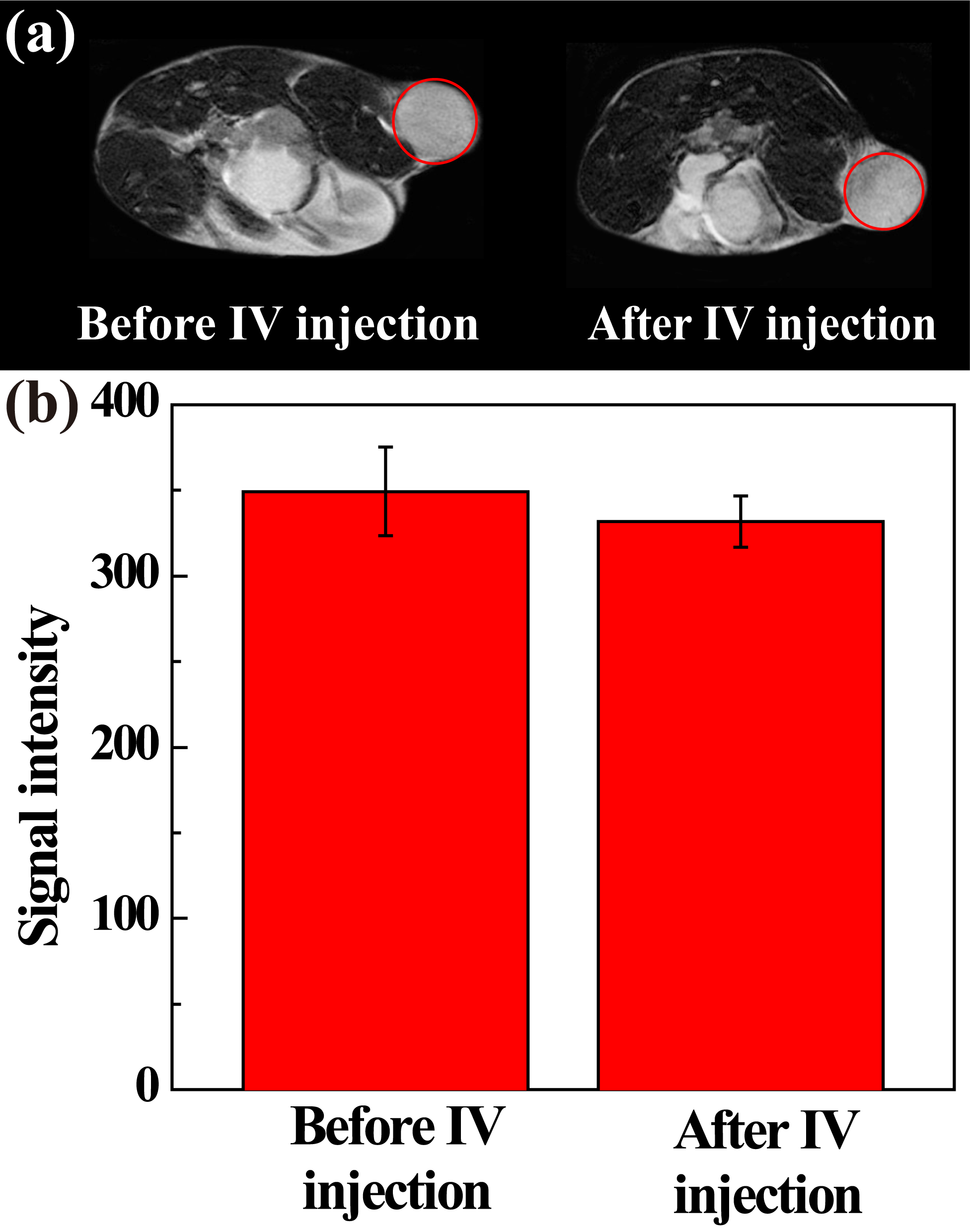
**

**Figure S11.** (a) T_2_-weighted MR images, (b) MR signal intensity of tumors in the FA blocking experiment before and at 6 h post IV injection of the Fe_3_O_4_@Au-PEI.Ac-PEG-FA NSs ([Fe] = 1.31 mM, 0.1 mL in PBS).

**
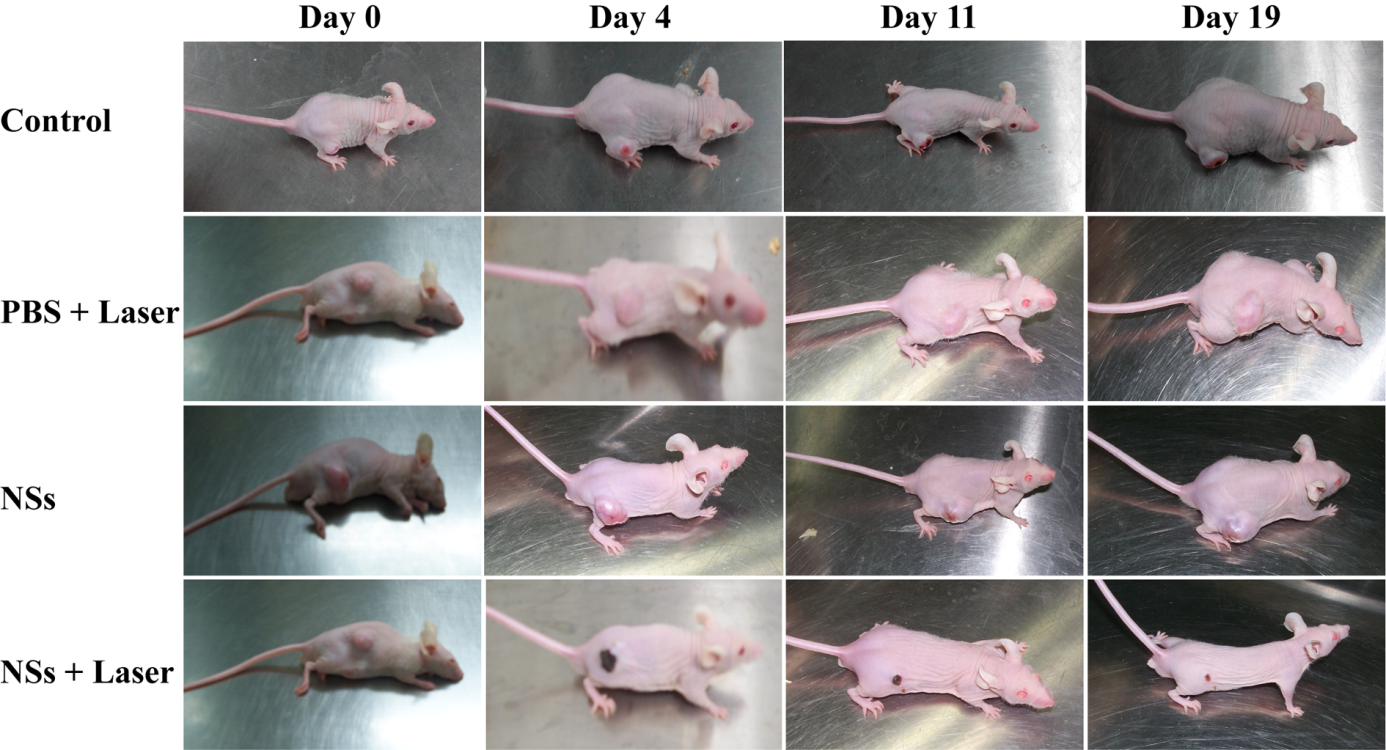
**

**Figure S12.** Photographs of the tumor mice under different treatments at different time periods.

**Figure S13.** Apoptosis rate of tumor cells after different treatments by quantification of the TUNEL-positive tumor cells in tumor sections.

**Table S1.** Zeta potential, hydrodynamic size, and polydispersity of the Fe_3_O_4_@Au-PEI, Fe_3_O_4_@Au-PEI-PEG-FA, and Fe_3_O_4_@Au-PEI.Ac-PEG-FA NSs. Data are provided as mean ± S.D. (n= 3).

| Materials | Zeta potential  (mV) | Hydrodynamic size (nm) | Polydispersity  index (PDI) |
| --- | --- | --- | --- |
| Fe_3_O_4_@Au-PEI | +31.4 ± 0.7 | 211.3 ± 2.1 | 0.34 ± 0.03 |
| Fe_3_O_4_@Au-PEI-PEG-FA | +28.3 ± 0.6 | 226.1 ± 3.6 | 0.33 ± 0.01 |
| Fe_3_O_4_@Au-PEI.Ac-PEG-FA | +14.4 ± 0.2 | 224.2 ± 4.9 | 0.37 ± 0.03 |
